# Tailorable Topological Multimode Nanolaser with Mutually Incoherent Modes

**DOI:** 10.1021/acsnano.5c22211

**Published:** 2026-06-29

**Authors:** Laura Yrjänheikki, Roman Calpe, Marek Nečada, Janne I. Heikkinen, Matias Koivurova, Antti J. Moilanen, Tommi K. Hakala

**Affiliations:** Center for Photonics Sciences, 163043 University of Eastern Finland, FI-80100 Joensuu, Finland

**Keywords:** nanolasers, plasmonic lattices, surface lattice resonances, topological photonics, coherence

## Abstract

We demonstrate a tailorable topological multimode nanolaser that supports simultaneous lasing in modes belonging to different topological classes and exhibiting no mutual phase correlation. Arrays of gold nanoparticles (NPs) with varying diameters were fabricated and embedded in a fluorescent dye gain medium, enabling the systematic investigation of the emergence and interplay between topologically trivial dipolar and topologically nontrivial quasi-bound-state-in-the-continuum (qBIC) modes. Angle- and wavelength-resolved measurements reveal single- and dual-mode lasing behavior, with far-field emission patterns and threshold characteristics strongly dependent on nanoparticle size. Spatial and temporal coherence characterization using interferometry shows mode-dependent coherence distributions across both the source plane and the far-field. We show that the relative contributions of the lasing modes can be tuned by adjusting the nanoparticle geometry, providing insights into the interplay between topologically distinct photonic modes and coherent light emission. Cross-correlation frequency-resolved optical gating measurements further resolve the temporal dynamics of the topologically trivial and qBIC modes, showing that these modes, having orthogonal polarizations at the sample plane, are mutually incoherent. This is in stark contrast to our previous results on topologically trivial superlattice modes, where shared excited-state molecular populations led to mode locking. The absence of mutual phase correlations is advantageous for applications requiring minimal crosstalk between lasing channels, such as multiplexed communication and multichannel sensing.

## Graphical abstract

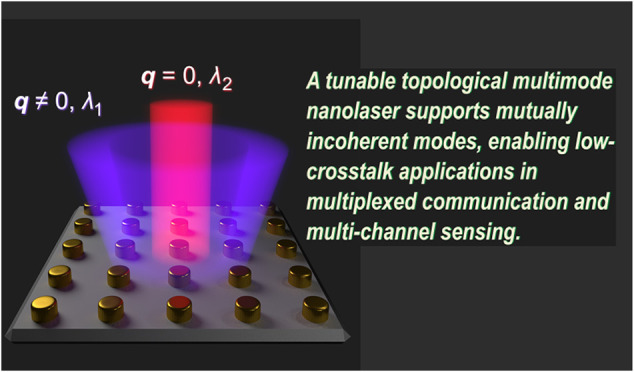

Topological photonics has become an important framework for achieving robust light transport and lasing, leveraging nontrivial band topology to protect optical modes against disorder and fabrication imperfections.^1,2^ Despite these advances, most topological lasers remain limited to single-mode operation or multimode configurations with enforced phase coherence, such as mode-locked systems.^3,4^ Although mutual phase correlations between lasing modes are advantageous for applications like ultrafast pulse generation through mode locking, their presence limits the ability to realize independent optical channels. Conversely, the absence of such correlations can be highly beneficial for developing flexible light sources that provide stable, crosstalk-free parallel outputs for next-generation photonic architectures.

Metallic nanoparticles (NPs) support localized surface plasmon resonances (LSPRs),^5^ which are characterized by strongly enhanced near fields.^6^ When arranged into a periodic array forming a plasmonic lattice, these nanoparticles can couple through in-plane radiation fields, giving rise to delocalized, dispersive surface lattice resonances (SLRs). These resonances originate from the hybridization of LSPRs with the diffracted orders of the lattice.^7–10^ Plasmonic lattices have been demonstrated to support modes with dipolar and quadrupolar character.^11,12^ When these lattices are combined with optically pumped gain media, such as fluorescent molecules, they can support lasing and condensation.^12–15^ The emission characteristics of such plasmonic nanolasers can be tailored by adjusting parameters of the lattice structure,^16^ including geometry,^17–20^ gain material,^21,22^ and nanoparticle properties.^23^

Plasmonic lattice nanolasers can generate ultrashort optical pulses^24^ and exhibit millimeter-scale spatial coherence.^25^ Picosecond-scale temporal coherence has been reported under femtosecond-scale pumping,^25,26^ while longer pumping pulses can extend coherence times from tens to hundreds of picoseconds.^27,28^ Notably, tuning the nanoparticle size alone can induce a transition from one-dimensional to two-dimensional spatial coherence,^29^ underlining the sensitivity of beaming properties to structural parameters. Beyond topologically trivial modes, interest has recently arisen in nanophotonic structures that support modes with nonzero topological charge, such as bound states in the continuum (BICs).

BICs have emerged as a powerful concept in nanophotonics, enabling optical modes with theoretically infinite lifetimes despite their spectral overlap with the radiation continuum.^30–32^ These modes arise from symmetry protection or destructive interference, which suppresses radiation to the far-field and leads to strongly confined resonances with extremely high cavity quality (*Q*) factors. Zhen et al. established a connection between BICs and the topological properties of photonic systems.^33^ They showed that optical BICs host vortex centers of polarization fields defined by a conserved quantity known as topological charge (*q*), which counts the number of 2π polarization windings around a closed loop in the momentum space.^33,34^ For topologically trivial modes *q* = 0 and for topologically nontrivial modes *q* ≠ 0. The discreteness of this charge renders BIC-derived modes inherently robust against small structural imperfections or disorder, providing a unique platform to enhance light–matter interactions. Ideal BICs cannot couple into external radiation and therefore do not appear in the far-field. However, in realistic systems, defects and finite-size effects, such as the sample’s edges, give rise to observable radiative quasi-BICs (qBICs).^35,36^ In periodic nanostructures, such as dielectric or plasmonic lattices, qBICs manifest as sharp resonances around specific symmetry points in momentum space. These modes typically produce a doughnut-shaped far-field pattern in which the bright emission arises from finite wavevectors surrounding the BIC momentum, while the exact BIC remains dark.^37^ Moreover, measuring polarization reveals a winding around this dark core, manifesting qBIC’s topological nature. Combined with their high *Q* factors and strong in-plane confinement, these features make qBICs attractive candidates for diverse photonic applications, such as low-threshold lasing and other nonlinear phenomena.^32,38,39^

Recently, (q)­BIC-based lasing and condensation have been reported across a variety of photonic and polaritonic systems,^40–46^ including plasmonic lattices,^12,36,47–50^ highlighting the ability to tailor the polarization and topological properties of BICs.^51^ Coherence properties of BIC-mode condensation in metasurfaces of silicon nanoparticles have been reported previously;^46^ however, the coherence and temporal dynamics of plasmonic lattice nanolasers supporting BICs remain unexplored. A deeper understanding of these features of topological nanolasers is essential for unlocking the full potential of BIC lasing and advancing ultrafast photonic technologies.

Here, we present an experimental study of the coherence properties and temporal dynamics of plasmonic lattice nanolasers supporting two modes belonging to distinct topological classes and having different polarization properties. Previous studies have demonstrated multimode lasing in topologically trivial modes using plasmonic lattices with supercells,^52^ rhombohedral particle symmetry,^53^ or multipatch lattice designs.^54,55^ Simultaneous lasing of multiple BIC modes has also been reported in square lattices of TiO_2_ nanoparticles.^56^ Our work represents an intermediate case, in which two topologically distinct modes—trivial (*q* = 0) and nontrivial (*q* ≠ 0)—coexist at zero wavevector in a simple square lattice. By systematically increasing the nanoparticle diameter, we demonstrate a transition from topologically trivial dipole-mode lasing to a combination of simultaneous dipole and qBIC lasing modes. This transition is accompanied by pronounced changes in both coherence properties and temporal dynamics. In particular, we show that the relative lasing intensities of these modes can be adjusted by structural design and that the modes are not mutually correlated.

The lack of phase correlations provides distinct advantages for optical information technologies that require minimal crosstalk between the optical components, modes, or channels within the system.^57–61^ Crosstalk can be suppressed or eliminated through various structural and modal design strategies, such as dedicated geometries^62–66^ or the use of higher-order waveguide modes.^67^ Mutually incoherent lasing channels, lacking a fixed phase relationship, inherently minimize crosstalk and interference between modes of different characters (e.g., polarization or topological class). This property enables stable, parallel optical outputs suitable for applications such as multiplexed communication, neuromorphic photonics, and multichannel sensing. In our system, the lasing modes with distinct topology and polarization characteristics are mutually uncorrelated, eliminating the need for additional crosstalk-suppression techniques and paving the way toward tailorable topological nanoscale light sources for integrated multichannel photonic devices.

Miniaturized topological multimode lasers represent a promising emerging technology because they combine four desirable features within a single platform: compactness, robustness, rich multimode dynamics, and additional degrees of freedom arising from their topological properties. These features may benefit wavelength-division-multiplexed optical communication, chip-scale spectroscopy, precision metrology, LiDAR, and topology-specific quantum photonics.

## Results and Discussion

### Plasmonic Lattices and Lasing Modes

The plasmonic lattice consists of a 150 × 150 μm^2^ array of periodically (*p*_*x*,*y*_ = 570 nm) arranged cylindrical gold nanoparticles (NPs), see Methods for details of the sample fabrication. These lattices support dispersive modes, SLRs, whose spectral and angular properties depend on the lattice geometry. We characterized their optical band structures using angle-resolved white-light transmission measurements without fluorescent dye molecules, see Supporting Information, Section S1, for the results. The ideal BIC mode is “dark” at zero incidence (wavevector **k** = 0), as it does not couple to the far-field. Without a gain medium, the spatial coherence of the SLRs is limited to around 60 μm, as obtained from the dispersion line width (2π/Δ*k*). However, when spatial coherence increases due to lasing, this dark mode can couple out to the far-field from the edges of the finite array as a qBIC.^12,35^ We note that, following common usage in literature,^68^ we refer to the system as a nanolaser because the plasmonic modes exhibit nanoscale vertical confinement, even though the lateral cavity dimensions are micrometer scale.

For lasing experiments, the lattice was overlaid with a gain medium composed of fluorescent IR-792 molecules dissolved in a benzyl alcohol (BA)–dimethyl sulfoxide (DMSO) solution. We use a concentration of 10 mM, which places the samples in the weak light–matter coupling regime.^15^ Lasing is triggered by optical pumping with a circularly polarized femtosecond-pulsed laser. Below the lasing threshold, the emission spectrum of the system is dominated by the spontaneous emission of the gain medium and shows the diffracted orders (Figure 1a, left). Just above the threshold (Figure 1a, right), the emission exhibits lasing on two modes, both located at zero wavevector (**k** = 0), i.e., orthogonal to the lattice plane (θ_*x*,*y*_ = 0). We employed a custom-built measurement setup, schematically shown in Figure 1b, to analyze the light emission from our samples. The emission was divided by beam splitters and directed to three types of measurement systems: (i) two configurations of wavefront-folding interferometers (WFIs) for analyzing coherence properties in the far-field and at the source plane, respectively; (ii) a spectrometer for obtaining angle-resolved far-field spectra; and (iii) a nonlinear crystal placed in front of the spectrometer, where the emission was combined with a delayed pump beam for cross-correlation frequency-resolved optical gating (XFROG) measurements of temporal dynamics. Further details of the setup and additional schematics are provided in the Supporting Information, Section S2.

**Figure 1:**
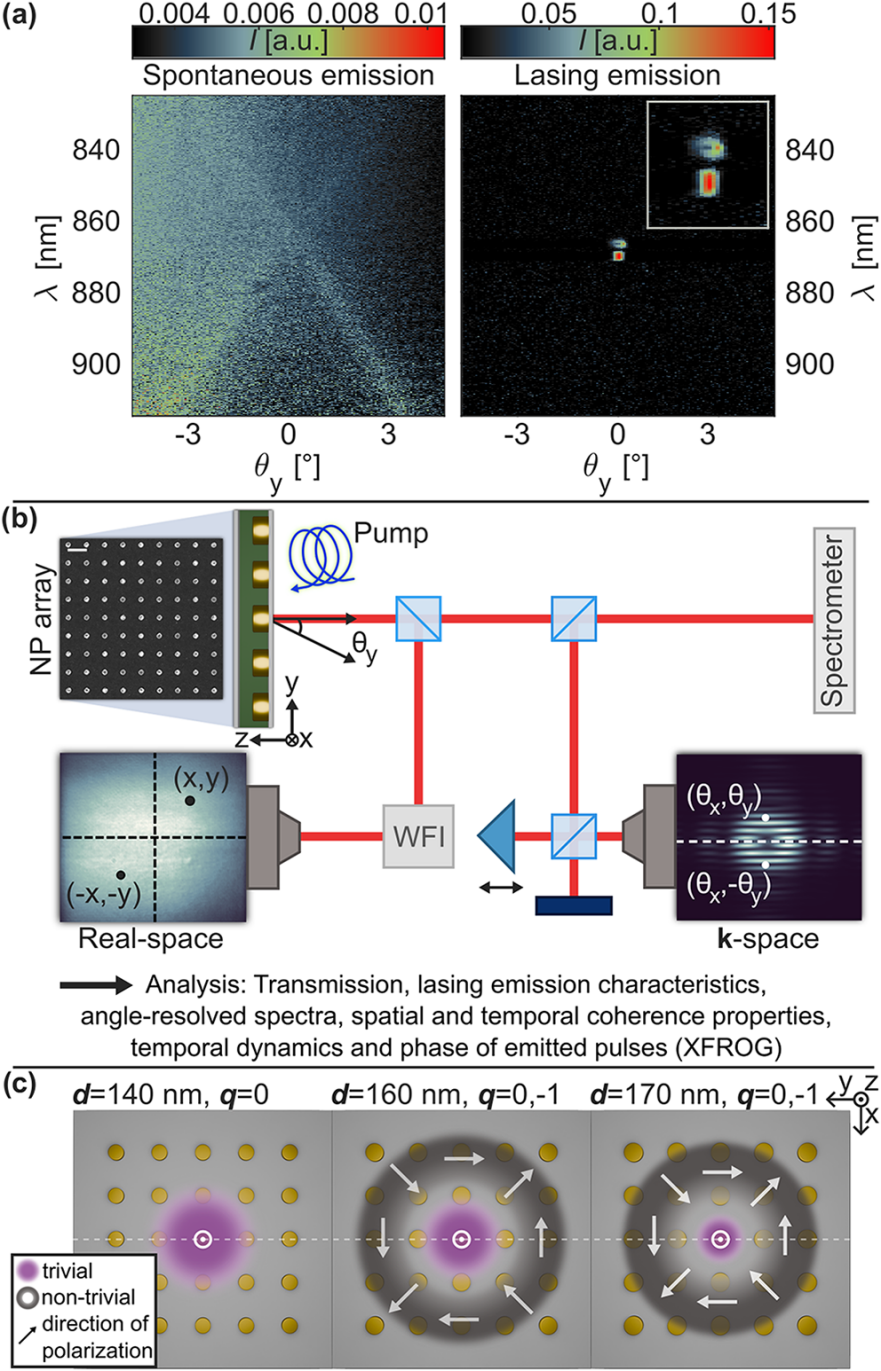
Schematics of the experimental setup and lasing modes of the samples. (a) Emission spectrum of the system below the lasing threshold (left) and above the threshold (right). The inset shows a magnified view of the lasing region. In both images, each row (wavelength) is normalized to its sum. The sample is a lattice with *d* = 160 nm nanoparticles (NPs). (b) Schematic of the measurements and one sample imaged with a scanning electron microscope (SEM). The sample is a periodic array of gold NPs forming a plasmonic lattice, overlaid with a solution containing fluorescent dye molecules. The white scale bar in the SEM image denotes the lattice constant (570 nm). A wavefront-folding interferometer (WFI) is employed to examine point-symmetric spatial correlations between positions (*x*, *y*) and (−*x*, −*y*) on the lattice plane (real-space image plane). Another WFI (*k*-space) folds the emitted beam along θ_*y*_ = 0°, enabling us to measure correlations between (θ_*x*_, θ_*y*_) and (θ_*x*_, −θ_*y*_). (c) Schematic of three lattices exhibiting different far-field emission properties as the *d* increases. The emitted lasing modes are associated with different polarization properties and topological charges of 0 (trivial, purple) and −1 (nontrivial, gray). White arrows and circles describe the polarization properties of the modes.

We systematically varied the NP diameter, *d* (140, 160, and 170 nm), within the lattice while keeping all other structural parameters fixed (Figure 1c). Changing *d* modifies both the far-field emission and the near-field distribution on the lattice plane.^29^ For small *d*, only a topologically trivial mode is present, characterized by a dipolar charge distribution at each nanoparticle and producing a dot-like pattern in the far-field. As *d* is increased, a topologically nontrivial qBIC mode emerges alongside the dipolar mode, manifesting as a quadrupolar charge distribution in the center of the lattice and as doughnut-shaped far-field emission pattern, as will be shown below. We confirmed the topological nature of the qBIC mode from the far-field emission by rotating a linear polarizer to different angles (see Supporting Information, Figure S3). The two-lobe pattern rotates in the opposite direction to the polarizer, while the topologically trivial mode shows no polarization winding (*q* = 0). This observation is consistent with previous reports for similar structures.^35,51^ In addition, we measured polarization-resolved far-field and real-space images of a similar sample where the qBIC mode dominated (see Supporting Information, Figure S4) to clearly see the full 2π rotation of the two-lobe pattern, corresponding to a topological charge of *q* = −1.^33^ A further increase in the particle diameter starts to favor lasing from the topologically nontrivial qBIC mode, suppressing the observable interplay between the modes (see Supporting Information, Section S4). This structural parameter tuning enables tailoring the threshold behavior and the relative emission intensities of the two topologically distinct modes.

### Lasing Characteristics

Measured angle- and wavelength-resolved emission as a function of pump fluence reveals the characteristic threshold behavior of our plasmonic lattice nanolaser (Figure 2a,b). At low pump fluences, the spectra are dominated by the broad spontaneous emission of the dye solution, which peaks at λ = 850 nm with a full width at half-maximum (FWHM) of approximately 50 nm (see Supporting Information, Section S5, for the spectra). As the pump fluence increases, the emission intensity rises sharply, and the spectral line width narrows significantly, marking the transition to the lasing regime. For the lattice with *d* = 140 nm, a sharp lasing peak emerges at λ = 878 nm (Figure 2a, left). In contrast, lattices with larger NP diameters exhibit multimode lasing. The *d* = 160 nm lattice shows peaks at λ = 866 nm and λ = 870 nm (Figure 2a, middle), while the *d* = 170 nm lattice lases at λ = 867 nm and λ = 870 nm (Figure 2a, right). Although the peak at 870 nm appears relatively weak on the chosen scale, the threshold curve in Figure 2b (bottom) confirms that it corresponds to lasing.

**Figure 2:**
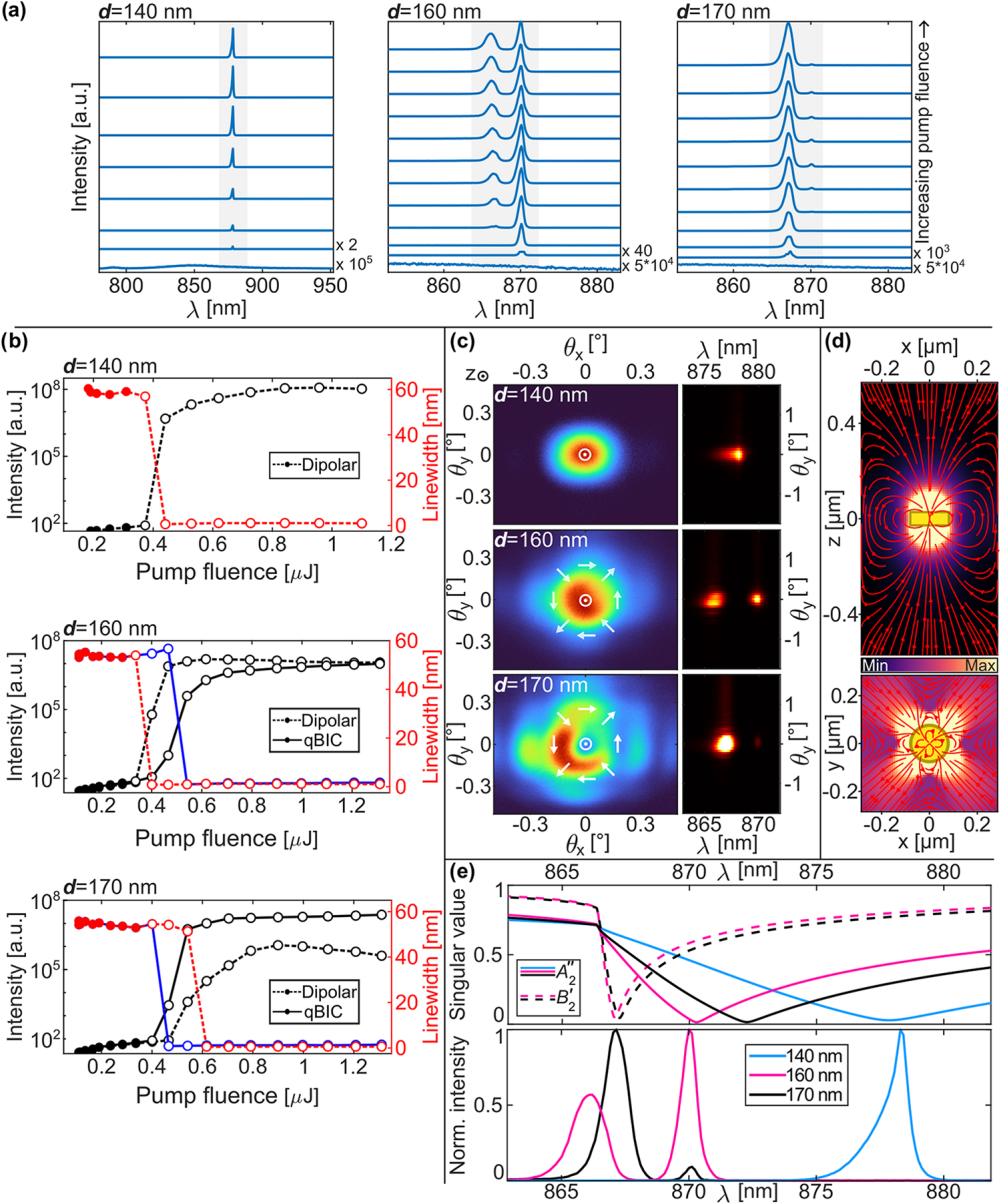
Characterizing lasing action of plasmonic lattices exhibiting single- and dual-mode emission. (a) Emission spectra at increasing pump fluence (from bottom to top). Spectra of dual-mode lasing are enlarged to distinguish the individual modes better. (b) Intensity of lasing emission (black lines, log-scale) and spectral line width (red and blue lines) of the lasing modes as a function of pump fluence. Unfilled data point markers correspond to spectra shown in (a). (c) Left: Emission patterns in the far-field of different samples and their polarization fields (white arrows and circles) for each lattice. Right: Lasing spectra and angular intensity distribution in θ_*y*_-direction, measured well above threshold. The middle and bottom images have the same wavelength axis. (d) Real part of the electric field patterns in the *xz*-plane (top) and inside a single unit cell in the *xy*-plane (bottom) corresponding to *A*_2_^″^ and *B*_2_^′^ lattice modes, respectively. The nanoparticle is located at the center of the unit cell. (e) Computed lowest-singular values of the *A*_2_^″^ and *B*_2_^′^ modes for lattices with different NP sizes (top). Enlarged and normalized lasing spectra for each lattice (bottom).

The threshold curves of the samples (Figure 2b) reveal a nonlinear increase in emission intensity by several orders of magnitude, accompanied by a line width reduction from around 50 to 0.5–2 nm. Interestingly, the two larger-diameter lattices exhibit distinct threshold behaviors. For *d* = 160 nm lattice, the longer-wavelength mode (λ = 870 nm) reaches threshold first, whereas for *d* = 170 nm lattice, the shorter-wavelength mode (λ = 867 nm) lases first. In both cases, the mode with the lower threshold also achieves the higher overall intensity.

These differences in lasing behavior are accompanied by distinct beaming patterns in the far-field (Figure 2c, left). Single-mode lasing produces a Gaussian-like beam profile with maximum intensity at the center, whereas two-mode lasing (*d* = 170 nm) results in a doughnut-shaped pattern. These pronounced changes arise from the fact that the two lasing modes belong to different topological classes. The dipolar mode (Figure 2d, top) is topologically trivial (*q* = 0) and features mainly *z*-oriented polarization, whereas the qBIC mode (Figure 2d, bottom) is topologically nontrivial (*q* = −1) and is characterized mainly by *x*- and *y*-polarized field components. At the *xy*-symmetry plane located midway between the particles, this polarization contrast becomes even more obvious: the trivial dipolar mode retains only a *z*-component, while the nontrivial qBIC mode exhibits exclusively in-plane *x*- and *y*-components. See Supporting Information, Section S6, for all electric field components. The spectral positions of the lasing modes agree, within reasonable accuracy, with the lowest-singular-value modes computed using the T-matrix method (Figure 2e), supporting their identification as topologically trivial dipolar *A*_2_^″^ and nontrivial *B*_2_^′^ modes (see Methods for description of the T-Matrix simulations).

We also investigated lasing in a plasmonic lattice using the FDTD method (Lumerical Inc.) combined with a built-in four-level two-electron gain material model. Simulations for a lattice with *d* = 160 nm confirmed lasing and the quadrupolar nature of the nontrivial *B*_2_^′^ mode. See Supporting Information, Figure S14 for the lasing emission, near-field profile, and population inversion dynamics.

Notably, since both modes exist at **k** = 0, they overlap in the far-field, which influences the shape of the resulting beam profiles. This contrast in topology manifests in the polarization fields of the far-field emission and in the relative intensities of the lasing modes, as evident in the angle-resolved spectra measured well above threshold (Figure 2c, right). Such variations in threshold and beaming behavior are expected to influence the spatial coherence at the lattice plane and in the far-field emission, as well as the temporal dynamics of the modes. In the following sections, we analyze the coherence properties and temporal dynamics of these samples in detail.

### Characterization of Coherence

We investigated spatiotemporal coherence both at the source plane (the real-space image plane of the lattice) and in the far-field (the *k*-space plane of the lattice) using custom-built WFIs^69–71^ (Figure 1b). The source-plane WFI measures spatiotemporal field correlations between point-symmetric positions (*x*, *y*) and (−*x*, −*y*) on the lattice, whereas the second WFI probes correlations between (θ_*x*_, θ_*y*_) and (θ_*x*_, −θ_*y*_) in the far-field emission. In both cases, we quantify coherence between the points **r** = (*x*, *y*) and −**r** = (−*x*, −*y*) by the complex degree of coherence μ­(**r**, −**r**) (see Supporting Information, Sections S2 and S6, for calculation and data processing details). This quantity can take values between −1 and 1, where the former denotes perfect anticorrelation and the latter stands for perfect correlation. Additionally, a value of 0 means no correlation, and in the following, we plot the absolute value |μ|.

#### Far-Field Coherence

Figure 3 illustrates the temporal coherence in the far-field for each lattice. For the lattice with *d* = 140 nm (Figure 3a), emission originates from a single dipolar mode, producing a uniform far-field beam profile as discussed above. At zero time delay (Δτ ≈ 0 s), distinct interference fringes span the entire beam cross-section (Figure 3a, top), indicating a high degree of coherence. To reduce noise, we averaged the measured |μ| over 0.07° in the θ_*x*_-direction (white dashed lines indicate the averaging region). The middle row shows the averaged spatial distribution of |μ| across the beam at several time delays, while the bottom row presents |μ| values along θ_*y*_ = 0°, crossing the beam center. At Δτ = 0 ps, |μ| exceeds 0.8, and comparable values in the upper and lower beam regions confirm uniform coherence across the cross-section.

**Figure 3:**
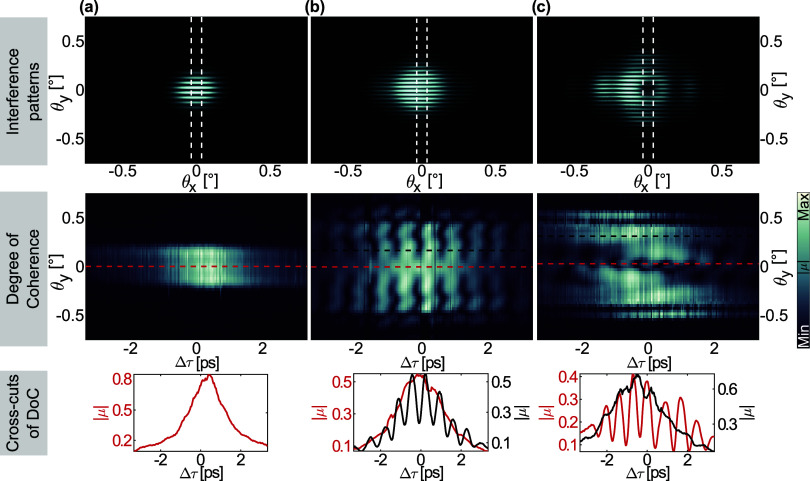
Coherence properties of lasing emission in the far-field from plasmonic lattices with NP diameters of (a) 140 nm, (b) 160 nm, and (c) 170 nm. Top row: Interference patterns measured using a WFI at zero time delay. White dashed lines denote the boundaries of the area used for averaging at each time delay. Middle row: Angular distribution of the averaged degree of coherence as a function of time delay, formed from the region marked in the top row. Each pixel represents |μ| averaged over 0.07° in the θ_*x*_-direction and 12 adjacent data points (0.028°) in the θ_*y*_-direction. Bottom row: Measured and noise-reduced |μ| along the red and black dashed lines shown in the middle row, corresponding to selected angular positions.

The qBIC mode appears alongside the dipolar mode for the lattice with *d* = 160 nm (Figure 3b). Its presence is evident in the interferogram (top row), where fringe visibility is highest at the center but slightly reduced toward the beam edges. The |μ| distribution across the beam cross-section (middle row) reveals a periodic beating pattern in specific angular regions where both modes exhibit equal intensity. To investigate this further, we compared two angular positions along the θ_*y*_ direction: one where beating is absent and one where it is evident. At θ_*y*_ = 0° (red dashed line), |μ| values indicate dominance of a single mode, as no beating occurs. In contrast, at θ_*y*_ = 0.16° (black dashed line), strong beating in |μ| confirms comparable contributions from both modes. The temporal beating period is *f*_b_ = 681 fs, defined as the time interval between successive maxima in |μ| oscillations. This value closely matches the inverse frequency difference between the two lasing modes, Δω^–1^ = 683 fs, obtained from the spectra in Figure 2c.

Figure 3c shows the far-field coherence properties of the lattice with *d* = 170 nm. The doughnut-shaped interferogram indicates that the qBIC mode dominates in intensity over the dipolar mode. Compared to the *d* = 160 nm lattice, the beating pattern in |μ| shifts toward the center of the emission profile (middle row), suggesting that both modes contribute equally around θ_*y*_ = 0° (red dashed line). At the upper angular position (θ_*y*_ = 0.31°, black dashed line), the absence of beating suggests that the qBIC mode dominates in that region. The beating period, *f*_b_ = 750 fs, again closely matches the inverse frequency separation of the modes, Δω^–1^ = 757 fs, obtained from the spectra in Figure 2c.

#### Source-Plane Coherence

Figure 4 shows the spatiotemporal coherence on the source plane, following the same NP diameters as in the far-field analysis (Figure 3). For the lattice with *d* = 140 nm (Figure 4a, top), distinct interference fringes span nearly the entire lattice, with maximum visibility at the center. The middle row displays |μ| values along the *y*-direction, averaged over 16 μm in the *x*-direction using the same procedure as in the far-field analysis. Around Δτ = 0 ps, the averaged |μ| exceeds 0.5, with comparable values across the vertical slice. The bottom row shows |μ| at *y* = 0 μm (red dashed line) in detail. The temporal evolution of |μ| closely resembles that observed in the far-field, confirming consistent coherence behavior across both domains.

**Figure 4:**
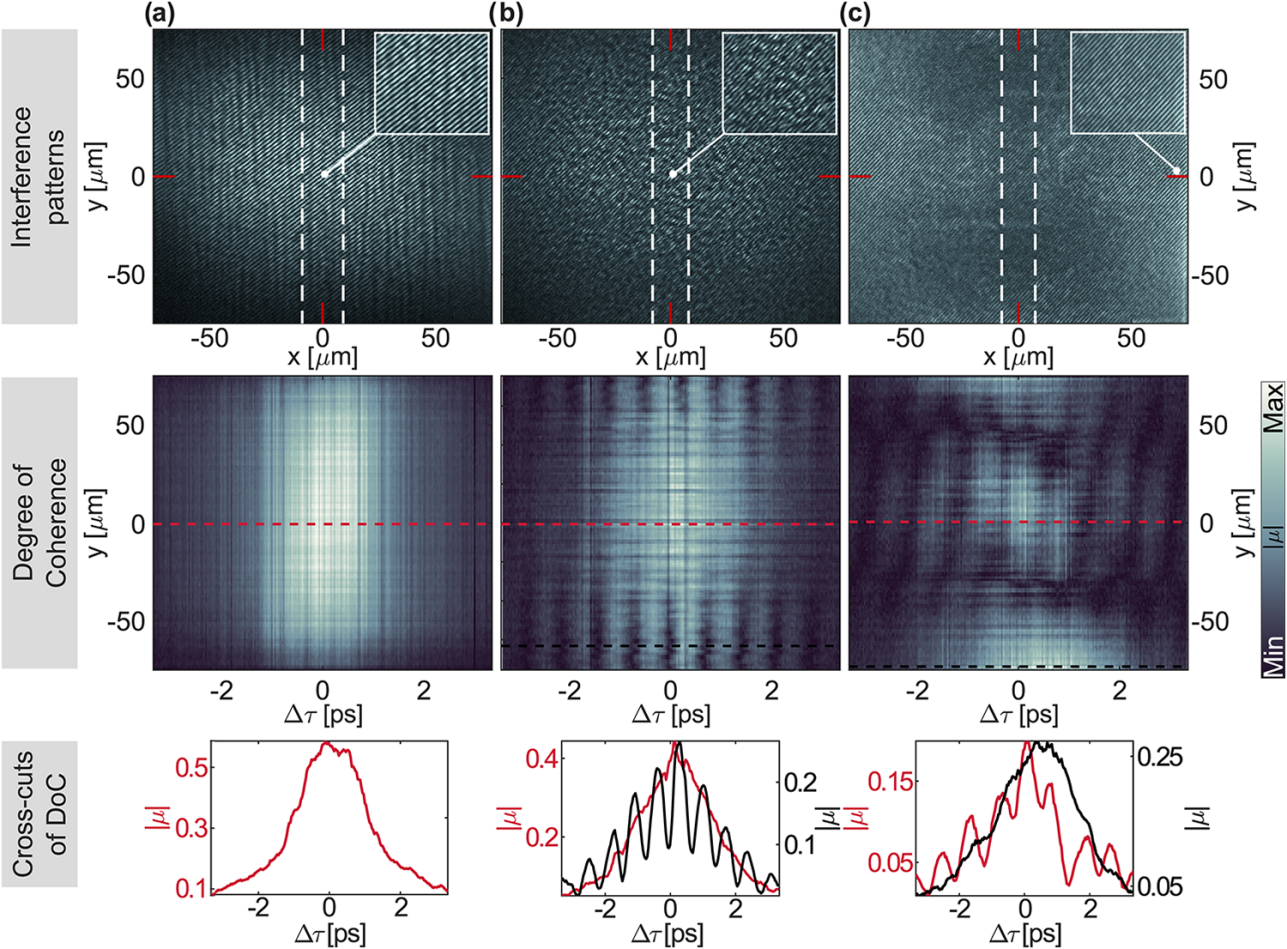
Coherence properties on the source planes of plasmonic lattices with NP diameters of (a) 140 nm, (b) 160 nm, and (c) 170 nm. Top row: Interference patterns measured using a WFI at zero time delay. White dashed vertical lines denote the boundaries of the averaging area, while red solid lines denote the lattice centers *x*, *y* = 0 μm. Insets show a zoomed-in view of interference fringes around marked regions. Middle row: Time-resolved degree of coherence, averaged over 16 μm in the *x*-direction and of 12 contiguous points (1.2 μm) in the *y*-direction. Bottom row: Measured and noise-reduced |μ| along the red and black dashed lines indicated in the middle row, corresponding to selected vertical positions on the lattice.

The presence of the qBIC mode in the *d* = 160 nm lattice (Figure 4b) makes the interference fringes less distinct in the central region compared to the *d* = 140 nm lattice. The averaged |μ| (middle row) exhibits a periodic beating pattern similar to that observed in the far-field coherence. Beating is evident at the upper and lower edges of the lattice, while the central region (*y* = 0 μm, red line) shows no oscillations. At *y* = −63 μm (middle and bottom rows, black line), the temporal beating has a period of *f*_b_ = 691 fs, closely matching both the inverse frequency difference and the beat frequency obtained from the far-field measurements. Averaging in the horizontal direction yields a comparable |μ| distribution (see Supporting Information, Section S6), indicating that the dipolar and qBIC modes contribute with equal intensity at the lattice edges.

The qBIC mode is more prominent in the lattice with *d* = 170 nm (Figure 4c), as indicated by pronounced interference fringes at the lattice edges, while the fringe contrast decreases in the regions above and below the lattice center. Compared to the *d* = 160 nm lattice, the periodic beating pattern in |μ| shifts toward the lattice center (*y* = 0 μm), while the outer edges (*y* = −74 μm) show no beating (middle and bottom rows). Consequently, the qBIC mode dominates near the lattice edges, whereas both modes contribute equally near the center over a span of approximately 60 μm. The temporal beating observed along the red dashed line has a period of *f*_b_ = 812 fs, which is slightly longer than the inverse frequency difference between the modes obtained from the far-field analysis.

These observations indicate that the topologically distinct lasing modes with distinct polarization states coexist and overlap in specific angular regions in the far-field and at certain spatial locations on the lattice source plane. However, the measurements described above provide information only about spatial correlations of the field. When multiple lasing modes are present, the system can host rich dynamical behavior, including temporal mode switching^72,73^ or mode locking between modes with comparable intensities, the latter resulting in a fixed phase relationship and temporal oscillations observable in the temporal dynamics.^55^ To investigate whether such dynamics and correlations exist in the present system, we analyzed the temporal evolution and phase relationships of the modes.

### Temporal Dynamics of the Modes and Phase Retrieval

We employed XFROG measurements to investigate the temporal dynamics and an XFROG retrieval algorithm to find phases of the emitted lasing modes.^74^ See Supporting Information, Section S2, for details on the XFROG measurement and processing the data. We measured sum-frequency (SF) signals emitted from each lattice. For this correlation analysis, we focused only on the lattice with *d* = 160 nm in more detail since the measured trace clearly showed two pulses, see Figure 5a,b. Measured SF signals for other lattices are presented in Supporting Information, Section S7. Notably, the SF signals from the lattice with *d* = 160 nm overlapped in both the temporal and spectral domains. These signals correspond to the qBIC mode (magenta dashed line) and the dipolar mode (black dashed line), with measured SF wavelengths of 414.7 and 416.2 nm, respectively. These values closely match the theoretical SF wavelengths of the lasing modes, calculated as 413.8 and 414.7 nm, respectively.

**Figure 5:**
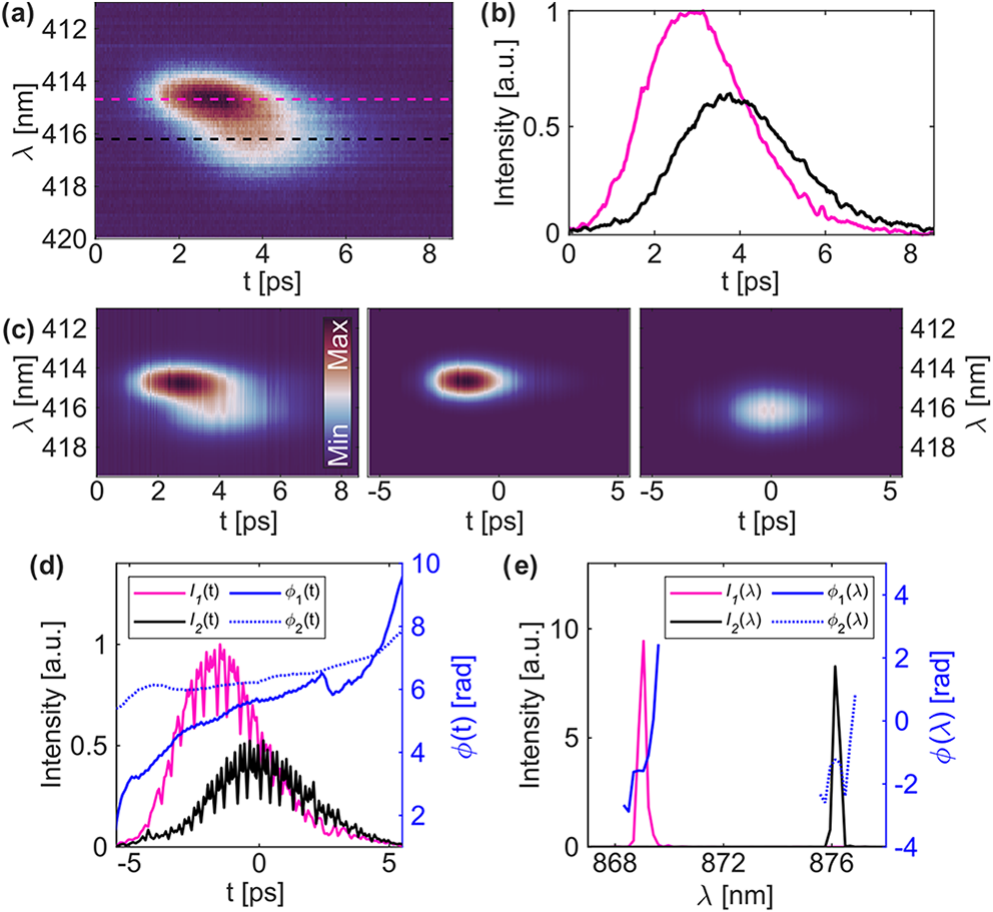
Temporal dynamics of the modes and phase retrieval. (a) Measured sum-frequency (SF) generation signal of a lattice with *d* = 160 nm. The signal shows two pulses that partially overlap in wavelength and time. The trace is integrated over relevant θ_*y*_-angles. (b) SF intensities along magenta and black dashed lines in (a). (c) Reproduced original trace represented as a sum (left) of Gaussian fits made for pulses at lower (middle) and higher (right) wavelength. The color scale is the same in each image. (d) Signal pulses and temporal phases obtained with the XFROG retrieval algorithm. (e) Retrieved spectral intensities and phases of both modes.

The emitted pulses overlapped closely in both the temporal and spectral domains. Moreover, since both modes exist at **k** = 0, it is not possible to separate them by analyzing the SF signal at different angles. When we processed the raw data using the double-blind XFROG retrieval algorithm, the result did not converge, and the FROG error remained high. Such failure to converge is a direct indication of low temporal coherence.^75^ This observation is consistent with the relatively low |μ|, which reached a maximum of approximately 0.5 for this NP size, also indicating a low degree of temporal coherence.^76^ However, this analysis alone does not reveal the origin of the low coherence. There are two possibilities: (i) one or both of the individual modes have low temporal coherence, or (ii) the individual modes are temporally coherent but mutually uncorrelated, such that the superposition of these modes features a low temporal coherence.

To investigate the origin of the low temporal coherence, we make an assumption that the modes are mutually uncorrelated, allowing the FROG trace to be divided into two subtraces, each containing the data of a single mode. When these are incoherently added, they reproduce the original trace (Figure 5c, left). We constructed the SF signals separately by fitting two Gaussian functions along the wavelength axis at each delay value in the measured data. Figure 5c (middle and right) shows the resulting traces, which were further adjusted to a suitable resolution before running the algorithm. The algorithm converged with trace-area-normalized FROG errors of 0.0838 (lower-λ mode) and 0.0971 (higher-λ mode). Typically, a value below 0.1 is considered indicative of good convergence. This, in turn, implies that the individual modes are temporally coherent and thus they must be mutually uncorrelated.

We further obtained the signal pulses and spectra of the modes along with temporal and spectral phases (Figure 5d,e). Notably, the retrieved signal pulses closely resemble the line profiles presented in Figure 5b. Both modes have a well-defined temporal and spectral phase, but there is no fixed phase relation between the modes from shot to shot (ϕ_2_ – ϕ_1_ ≠ constant). That is, the modes are indeed mutually uncorrelated. The streaks visible in the pulse profiles were caused by the fitting procedure, removing them would have further improved the FROG error. However, we intentionally performed the retrieval with minimal noise reduction to ensure that the subtraces accurately represent the original trace. Phase retrieval of the FROG traces, combined with the fact that the measured trace can be reconstructed from the subtraces, confirms that the two modes are not mutually phase-correlated. This demonstrates the absence of phase locking between the topologically trivial dipolar and topologically nontrivial qBIC lasing channels. Furthermore, if the modes were phase-locked, one would expect to observe an oscillating pattern indicating temporal beating directly in the measured trace, as observed in our previous work where multiple (topologically trivial) lasing modes were phase-locked.^55^

## Conclusions

In conclusion, we have demonstrated a tailorable multimode nanolaser that enables simultaneous lasing in modes belonging to different topological classes and having different polarization properties while exhibiting no mutual phase correlation. We performed a comprehensive investigation of the temporal coherence and dynamics of plasmonic lattice nanolasers operating either in a single dipolar mode or in two topologically distinct modes (dipolar and qBIC). Lasing was characterized in three lattice configurations exhibiting single- and dual-mode emission. By increasing the NP diameter, we observed pronounced changes in coherence properties in both the far-field emission and the source plane.

Furthermore, XFROG measurements were employed to reveal the temporal dynamics of the modes. Interestingly, although the two lasing modes coexist within the same lattice, their outputs exhibit no mutual phase correlation. This behavior contrasts with our previous results on plasmonic superlattices, where strong phase correlations between different lasing modes led to modulation of the lasing signal on the 100 fs scale.^55^ A plausible explanation for the different behavior is that the modes in the present work belong to distinct topology classes and have different polarization, whereas in ref 55 the lasing modes shared the same topology. We note that topology itself may not be a sufficient predictor of whether modes will or will not correlate. We believe that even modes with different topologies may in some cases exhibit phase correlations, provided that their polarization properties are sufficiently compatible or allow coupling. In our samples, the strong polarization orthogonality appears to prevent such coupling and thus provides a plausible route to the observed absence of phase correlations. Verifying this hypothesis calls for dedicated theoretical efforts and carefully controlled experiments on samples exhibiting lasing across various topological classes and distinct polarization properties, providing strong motivation for follow-up investigations.

Beyond the independent lasing behavior, an intriguing aspect of our results is the ability to tune the relative intensities of the lasing modes by adjusting the NP diameter. These findings pave the way for designing topologically robust multimode nanolasers with tailorable coherence and emission characteristics. Future work could extend to electrical control and pumping of such lattices,^77,78^ enabling integration with on-chip photonic circuits and leading to potential applications in multiplexed optical communications, neuromorphic photonics, and multichannel sensing.

## Methods

### Sample Fabrication

Gold nanoparticle arrays were fabricated using electron-beam lithography (Raith EBPG5000 + ES, 100 kV) and lift-off. A poly­(methyl methacrylate) (PMMA) resist was spin-coated onto the substrate, patterned by electron-beam exposure, and developed. Gold was deposited through thermal evaporation, followed by lift-off in acetone to define the arrays. The size of each lattice was 150 × 150 μm^2^. The lattice period was *p*_*x*,*y*_ = 570 nm and the height of each particle was 50 nm. For optical band structure measurements (see Supporting Information, Section S1), the lattices were combined with a liquid solution of BA and DMSO (2:1 volume ratio). For lasing experiments, organic fluorescent IR-792 molecules were dissolved in this solution at a concentration of 10 mM.

### Experimental Setup

To trigger the lasing action, we employed a circularly polarized femtosecond-pulsed laser (792 nm, 150 fs, 300 Hz) to optically pump the fluorescent molecules. The pump beam was incident at 45° to the lattice, forming an elliptical excitation spot of approximately 0.077 mm^2^, fully covering the lattice. The sample plane was imaged using a 10× microscope objective and a lens, with an aperture positioned at the image plane. Another lens was placed after the aperture, which projected the *k*-space image of the lasing emission onto the spectrometer. With beam splitters, the emission was directed to WFIs. In both WFI configurations, computer-controlled translation stages precisely varied the optical path difference between the interferometer arms, enabling accurate time-delay measurements. From the processed data, we extracted the degree of coherence (|μ|) at both in the far-field and at the lattice plane. For XFROG measurements, an additional beam splitter, a nonlinear crystal, and a low-pass filter were incorporated into the setup. A detailed schematic, data analysis procedure, and description of the measurement setup are provided in the Supporting Information, Section S2.

### T-Matrix Simulations

We utilized T-matrix simulations^79,80^ to identify the lasing modes in the system. The modes are given by the solutions of the multiple-scattering problem *M*(ω, **k**)*f* ≡ (*I* – *T*(ω)*S*(ω))*f* = 0, where *I* is the identity matrix, *T*(ω) is the T-matrix characterizing the scattering properties of individual NPs in the spherical wave basis, *S*(ω, **k**) is the translation operator describing spherical wave propagation between NPs in an infinite lattice, and *f* is the vector of induced multipole coefficients. The solution to the mode problem exists when the matrix *M*(ω) is singular, that is, when it has a zero singular value—the frequencies of the prospective modes (for a given wavevector **k**) are then found as the minima of the lowest-singular value of *M*(ω, **k**). Moreover, the *D*_4*h*_ point group symmetry of the system enables transforming the matrix *M*(ω) into a symmetry-adapted basis and solving the problem for each irreducible representation separately, as done in Figure 2e.

## Supplementary Material



## Notes

Transmission spectra; details of the setup and measurements; polarization-resolved emission; measurements of a sample with *d* = 180 nm; spontaneous emission of the gain medium and spectra below lasing threshold; electric fields of identified lattice modes computed with T-matrix method; processing of spatiotemporal coherence data; additional measured FROG traces; and FDTD simulations of lasing in an infinite plasmonic lattice (PDF)
