# Supplementary material for: Tailorable Topological Multimode Nanolaser with Mutually Incoherent Modes
Source: ACS Nano. 2026 Jun 29;20(27):19191–201. doi: 10.1021/acsnano.5c22211 (PMC13374383; doi:10.1021/acsnano.5c22211)
Supplement: Supplementary file 1 [file nn5c22211_si_001.pdf]

Supporting Information: Tailorable Topological Multimode Nanolaser with Mutually Incoherent Modes

Laura Yrjänheikki[1], Roman Calpe[1], Marek Nečada[1], Janne I. Heikkinen[1], Matias Koivurova[1], Antti J. Moilanen*[1], and Tommi K. Hakala*[1]

[1]Center for Photonics Sciences, University of Eastern Finland, FI-80100 Joensuu, Finland

*Email: tommi.hakala@uef.fi, antti.moilanen@uef.fi

List of contents:

Section S1. Transmission spectra
Section S2. Details of the setup and measurements
Section S3. Polarization-resolved emission
Section S4. Measurements of a sample with $d = 180$ nm
Section S5. Spontaneous emission of the gain medium
Section S6. Electric fields of identified lattice modes computed with T-matrix method
Section S7. Processing of spatiotemporal coherence data
Section S8. Additional measured FROG traces
Section S9. FDTD simulations of lasing in an infinite plasmonic lattice

Section S1. Transmission spectra

Figure S1 shows measured angle-resolved transmission spectrum of each sample investigated. The redshift of the band-edge is evident when nanoparticle diameter increases.

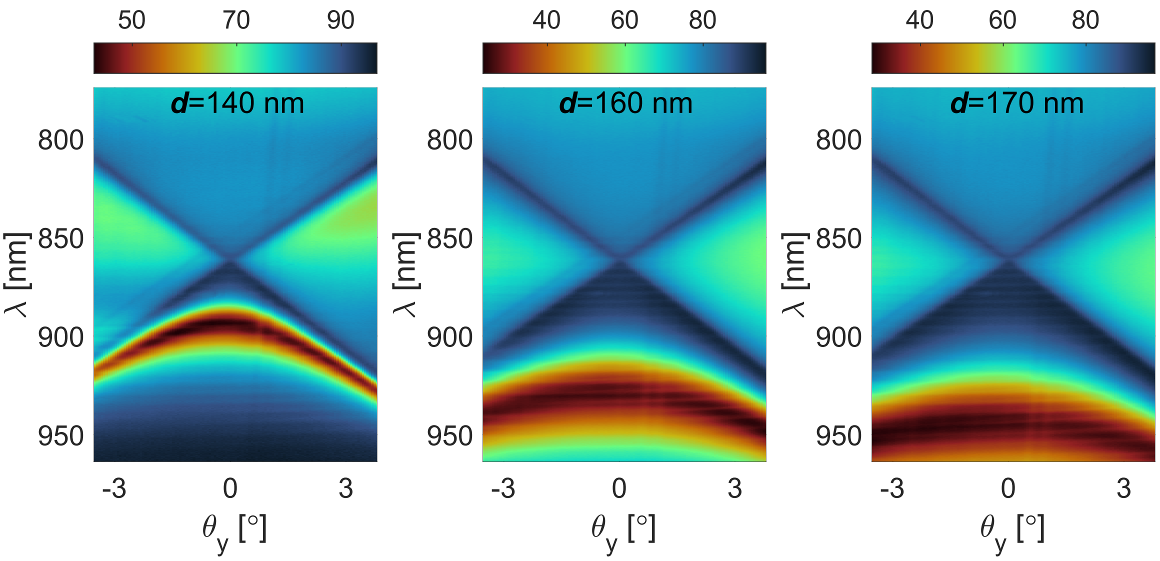

Figure S1: Measured TE-polarized transmission spectrum of each sample. Colorbar represents transmission (%).

Section S2. Details of the setup and measurements

Detailed description of the measurement setup is shown in Fig. S2. Each measurement type described in the main manuscript was possible to conduct within the setup with minor adjustments.

Lasing action from the plasmonic lattices was induced with optical pumping, represented as green line in Fig. S2. The laser used for the pumping was Pharos PH2 Femtosecond Laser System with Orpheus-HE High Energy Optical Parametric Amplifier, both by Light Conversion. Before the pumping beam entered the system, its power was controlled with a half-wave plate (HWP) mounted on a motorized stand, followed by a stationary polarizer (P1). Beam splitter (BS1) divided the beam into two, the first part going towards the plasmonic lattice and the second part being a reference pulse in the XFROG measurements (see details below). A singlet lens (L1) focused the pumping beam. A quarter-wave plate (QWP) in front of the sample modified the beam's polarization state to circular state.

Red line in Fig. S2 represents the emission from the system (also transmitted light, see details below). A 10× microscope objective (OBJ) magnified the emission, equipped with a high-pass filter to extract the scattered pump. The following doublet lens (L2) with a focal length of 200 mm acted as a collimator. A linear polarizer (P2) was optional and it was inserted for polarization-resolved analysis. An aperture (A) limited undesired light to pass through and also marked the boundaries of detection area on the sample. The following Fourier lens (L3) with a focal length of 100 mm projected the k-space image to the spectrometer. Beam splitters (BS3 and BS4) guided the emission to wavefront-folding interferometers, see details of them below.

To recover the optical band structure of the lattices in transmission measurements, a white light source (LS) with a diffuser was positioned behind the sample. The pumping path was omitted, but otherwise the setup was the same as in the lasing experiments. With linear polarizer, it was possible to measure TE- and TM-polarized band structures.

The blue line represents the path of the reference pulse in the XFROG measurements. The path was blocked in other measurements. The reflected beam from the beam splitter (BS1) propagated through a delay path consisting of two mirrors on motorized stage (outlined with black dashed rectangle) and passed through a half-wave plate. Beam splitter (90:10, BS2) was placed into the setup only in XFROG measurements to guide the reference pulse towards a nonlinear crystal (NLC). Beam splitters BS3 and BS4 were removed to collect the maximum intensity of the emission. The NLC, which was 2 mm thick β-Barium Borate, generated a sum-frequency (SF) signal from sample's emission and reference pulse as $\omega_{\mathrm{SFG}} = \omega_{\mathrm{em}} + \omega_{\mathrm{ref}}$, which was recorded with the spectrometer. A low-pass filter was placed after the NLC to prevent the second-harmonic signal of the sample's emission to propagate to the spectrometer.

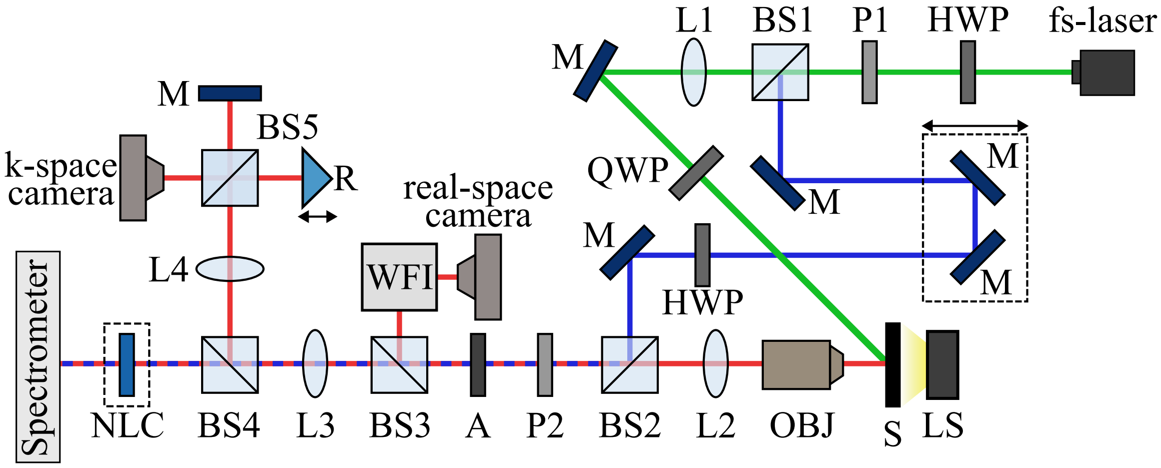

Figure S2: Schematic of the measurement setup.

The spectrometer used was Andor Kymera 193i with Andor Newton 970 EMCCD. The k-space image was projected on the entrance slit of the spectrometer, which enabled measuring angle-resolved spectrum in y-direction. Spectra were recorded with Andor Solis software. Background signal was measured before collecting the spectrum and every time a component was added or adjusted in the emission path. The exposure time was $0.3 - 0.5$ seconds for lasing measurements. In the transmission measurements, a reference signal (i.e. 100 % transmission) was measured in addition to the background signal. The transmission was recorded with an exposure time of 0.3 seconds and with accumulation, totaling 50 signals. In the XFROG measurements, the exposure time was one second and the accumulation totaled 10 signals. The reference pulse's path length was adjusted with computer-controlled stage, and the step size was set as 10 μm (33 fs in time).

For lasing and transmission measurements, neutral density filter was positioned in front of the spectrometer's entrance slit to prevent saturation. Neutral density filters were taken into account in the Fig. 2 of the main manuscript showing linewidth and threshold of the lasing modes. The filter limits a fraction of the incident light to transmit according to $\frac{I}{I_0} = 10^{-d}$, where I is the transmitted intensity, I_0 is the incident intensity, and d is the filter's optical density. The fluence of the pumping beam was measured with a pulse meter (PD10-C and Starbright by Ophir Photonics), which was

placed before beam splitter BS1. Beam splitters (BS1, BS3, and BS4) were also taken into account when calculating the sample's emission intensity and pumping fluence arriving to the sample.

Wavefront-folding interferometers and degree of coherence

We utilized two configurations of wavefront-folding interferometers (WFIs) [1–3] in our measurement setup to analyze spatiotemporal coherence properties and the mode interplay. The WFI for the far-field analysis contains (see Fig. S2) a beam splitter (BS5), mirror, retroreflector (R), and a recording camera. The retroreflector was mounted on a motorized stage which was controlled to adjust the time delay between interferometer arms. The retroreflector flips the field along horizontal axis, and enabled us to measure correlations between angles (θ_x, θ_y) and $(\theta_x, -\theta_y)$. Thus, it was reasonable to take a vertical slice for inspection (see details of data analysis in Section S6).

The custom-built real-space WFI folds the light field along horizontal and vertical axes, resulting a field overlap of point-symmetrical positions (x, y) and $(-x, -y)$ on the lattice plane. This WFI consist of six plane mirrors and two beam splitters. In both WFI configurations, the recording cameras were FLIR Grasshopper 3's (GS3-U3-32S4M-C).

Spatial coherence can be interpreted as correlations between light fields at two arbitrary spatial points, $\mathbf{r}_1$ and $\mathbf{r}_2$, at a chosen plane. It can be measured by detecting the visibility of the interference fringes. In both WFIs, the intensity at the detector can be defined by summing the fields that propagate in the individual arms as

$$I_D(\mathbf{R}; t) = |E(\mathbf{r}_1; t_1) + E(\mathbf{r}_2; t_2)|^2, \tag{1}$$

where $E(\mathbf{r}_i, t_i), i = 1, 2$, is the complex analytic representation of the field in the space-time domain, t denotes time, and $\mathbf{R} = (x, y)$ is the spatial coordinate at the detector. The above equation can be written as

$$I_D(\mathbf{R}; t) = I(\mathbf{r}_1) + I(\mathbf{r}_2) + 2\Re\{\langle E^*(\mathbf{r}_1; t_1)E(\mathbf{r}_2; t_2)\rangle\}, \tag{2}$$

where I is the time-integrated intensity, asterisk denotes the complex conjugate, and $\Re$ is the real part of the field. The angle brackets denote time or ensemble averaging, and in the context of Eq. 2, we consider time average. In the space-time domain, the spatial coherence properties of the field are described with the time-integrated mutual coherence function (MCF)

$$\Gamma(\mathbf{r}_1, \mathbf{r}_2; \Delta t) = \langle E^*(\mathbf{r}_1; t)E(\mathbf{r}_2; t + \Delta t)\rangle, \tag{3}$$

where $\Delta t = t_2 - t_1$ is the time difference between two copies of the incoming field. Inserting this into Eq. 2, we get

$$I_D(\mathbf{R}; t) = I(\mathbf{r}_1) + I(\mathbf{r}_2) + 2\Re\{\Gamma(\mathbf{r}_1, \mathbf{r}_2; \Delta t)\}, \tag{4}$$

In the space-frequency domain, the spatial coherence of the field is characterized with the cross-spectral density function (CSD)

$$W(\mathbf{r}_1, \mathbf{r}_2; \omega) = \langle E^*(\mathbf{r}_1; \omega)E(\mathbf{r}_2; \omega)\rangle, \tag{5}$$

where ω is the frequency of the field and now the angle brackets denote ensemble averaging. By setting $\mathbf{r}_1 = \mathbf{r}_2 = \mathbf{r}$ in the above equation, we obtain the spectral density $S(\mathbf{r}, \omega) = W(\mathbf{r}, \mathbf{r}, \omega)$ of the field, which describes field's average energy density at a specific point and frequency. A connection between CSD and time-integrated MCF is found by the Wiener-Khintchine theorem as

$$\Gamma(\mathbf{r}_1, \mathbf{r}_2; \Delta t) = \int_0^\infty W(\mathbf{r}_1, \mathbf{r}_2; \omega) \exp{(-i\omega\Delta t)} \mathrm{d}\omega. \tag{6}$$

Thus, we can replace the term containing the time-integrated MCF in Eq. 4 with the above relation. Furthermore, setting the time difference to zero and using the definition of CSD, we obtain

$$I_D(\mathbf{R}) = I(\mathbf{r}_1) + I(\mathbf{r}_2) + 2\Re\Big\{\int_0^\infty \langle E^*(\mathbf{r}_1;\omega)E(\mathbf{r}_2;\omega)\rangle \mathrm{d}\omega\Big\}. \tag{7}$$

The complex degree of coherence in the space-frequency domain is a normalized quantity defined as

$$\mu(\mathbf{r}_1,\mathbf{r}_2;\omega) = \frac{W(\mathbf{r}_1,\mathbf{r}_2;\omega)}{\sqrt{S(\mathbf{r}_1;\omega)S(\mathbf{r}_2;\omega)}} = |\mu(\mathbf{r}_1,\mathbf{r}_2;\omega)|\exp\left[i\alpha(\mathbf{r}_1,\mathbf{r}_2;\omega)\right], \tag{8}$$

where α is the phase of the complex degree of coherence. Following from the Wiener-Khintchine theorem, the temporal intensity and the spectral density are related by

$$I(\mathbf{r}) = \int_0^\infty S(\mathbf{r};\omega)\mathrm{d}\omega. \tag{9}$$

Using these equations (Eqs. 8 and 9), we end up with the following equation for the fringe pattern measured at the detector

$$I_D(\mathbf{R}) = I_1(\mathbf{R}) + I_2(\mathbf{R}) + 2\sqrt{I_1(\mathbf{R})I_2(\mathbf{R})}|\mu(\mathbf{r})|\cos\left[\alpha(\mathbf{r})\right], \tag{10}$$

from which we can determine the absolute value of degree of coherence. In Eq. 10, we have denoted $I(\mathbf{r}_i) = I_i(\mathbf{R})$. [3, 4]

The WFIs capture four images: one image with both interferometer arms open to acquire the interference fringes ($I_D(\mathbf{R})$), two with only single arm open ($I_1(\mathbf{R})$ and $I_2(\mathbf{R})$), and one image of the background noise with both arms closed. The images recorded with only one of the arms open give the spatial intensity of the field. Before further processing of the measured data, the recorded background noise is removed from every other measured image.

The procedure for the measurements with both WFIs was the same as in [5]; the temporal evolution of coherence was obtained by adjusting the optical path length of one arm in the WFIs and measuring spatial coherence. The step size was set to 10 μm and converted into time for data processing.

Section S3. Polarization-resolved emission

Polarization-resolved spectra and beam profiles in the far field of a $d = 160$ nm sample are shown in Fig. S3. The angles of the polarizer denoted on top of the figure are with respect to θ_y-axis. Note that both lasing signals spatially overlap in the far field. Topological charge corresponds to the number of 2π polarization windings around the BIC momentum, given by $q = \frac{1}{2\pi}\oint_C d\mathbf{k} \cdot \nabla_{\mathbf{k}}\phi(\mathbf{k})$. In the equation, C is a closed loop formed around the BIC momentum and $\phi(\mathbf{k})$ is the angle of the polarization vector. [6] We can conclude that the qBIC mode has a topological charge of -1, while the dipolar mode is topologically trivial, that is, the mode shows no polarization winding. [7, 8]

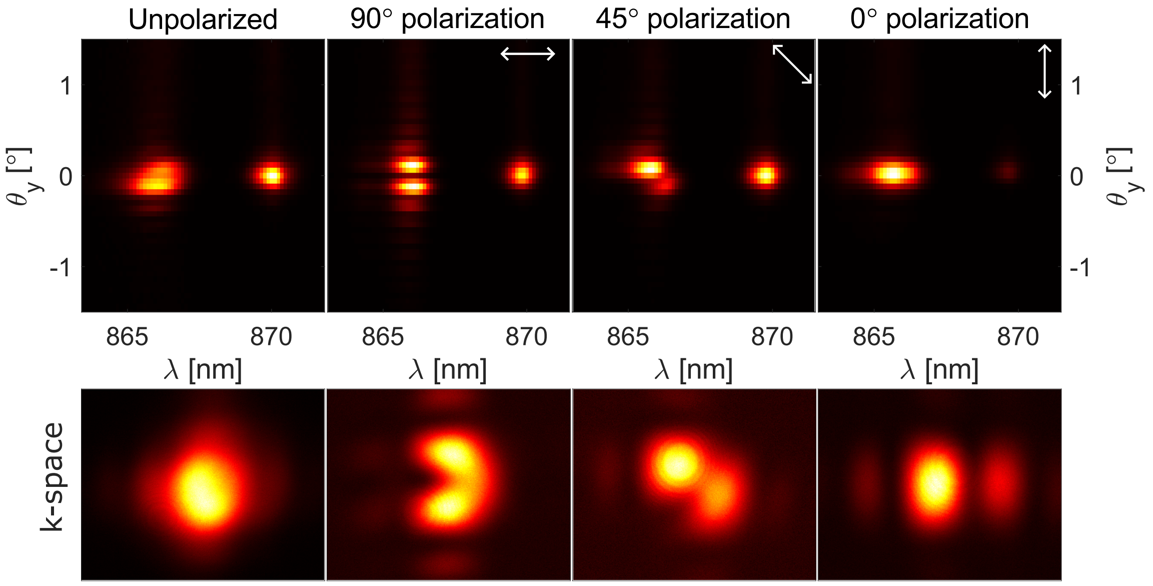

Figure S3: Polarization-resolved spectra (top row) and beam profiles (bottom row) of the far-field emission of the sample $d = 160$ nm. Arrows denote the orientation of the linear polarizer.

Figure S4 shows polarization-resolved far-field and real-space images of a sample in which the qBIC mode overwhelmingly dominates. Polarization winding around the dark core in the far field is apparent. Bright emission lobes rotate to the opposite direction than the linear polarizer, denoting topological charge of -1.

In real space, the center of the lattice exhibits a quadrupolar charge distribution. While the ideal BIC mode is non-radiative in the infinite lattice, since the quadrupolar charge is present everywhere, finite-size effects lead to the emergence of induced dipole moments that become strongest at the lattice edges. These dipoles break the perfect destructive interference and enable out-coupling of the qBIC mode into the far field. [7, 9]

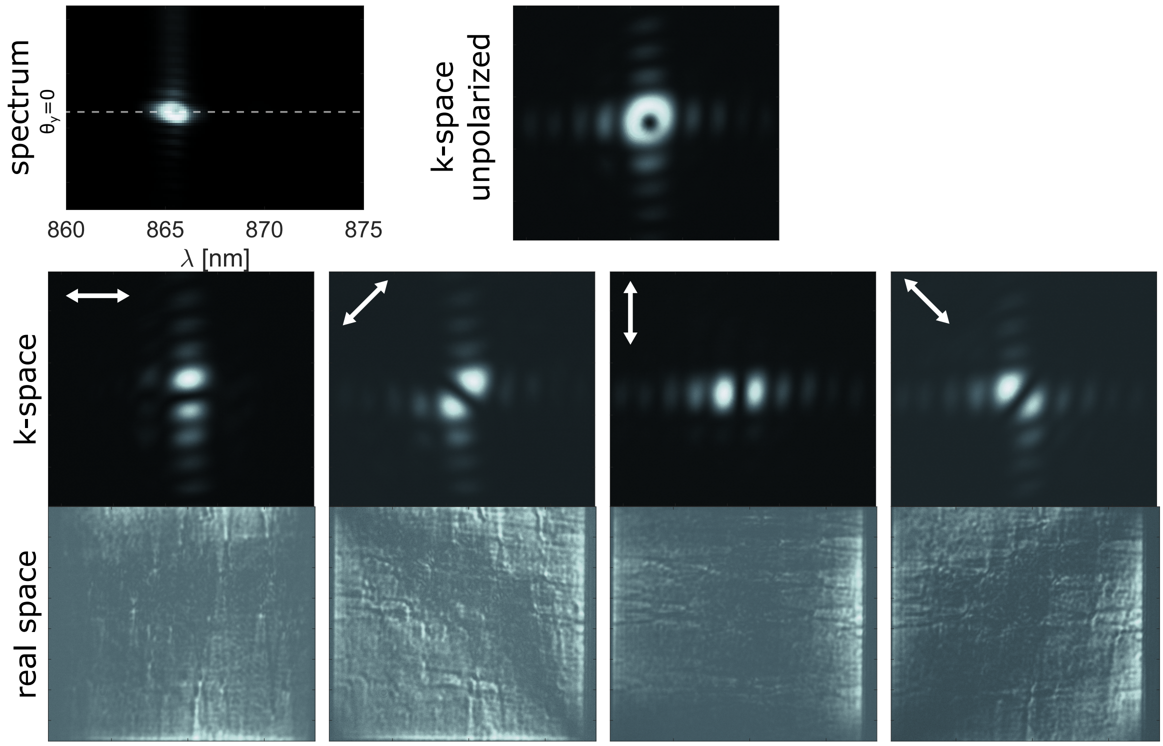

Figure S4: Unpolarized spectrum and polarization-resolved measurements of a sample where the qBIC mode dominates.

Section S4. Measurements of a sample with $d = 180$ nm

Figure S5 shows angle-resolved spectrum, coherence, and XFROG measurements of a sample in which the qBIC mode dominated. From the figures it is clear that the interplay (temporal beating in the degree of coherence) between the modes is not observable anymore.

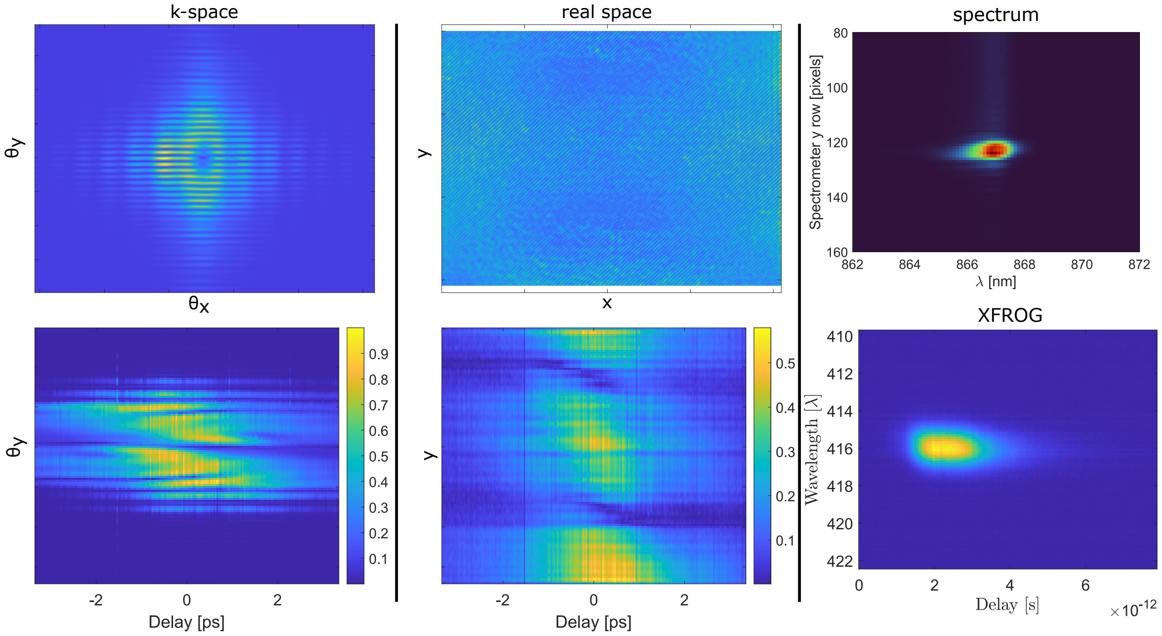

Figure S5: Coherence and XFROG measurements of a sample with $d = 180$ nm. Top row shows a single interferogram around zero delay while bottom row shows the degree of coherence as a function of time, calculated as shown in Section S7.

Section S5. Spontaneous emission of the gain medium and spectra below lasing threshold

Figure S6 shows the spontaneous emission of the gain medium peaking at $\lambda = 850$ and the femtosecond pumping laser signal at $\lambda = 792$ nm. The spectrum was recorded during the threshold measurement, the lattice being below the gain medium. Figure S7 shows all recorded below-threshold spectra and the corresponding pump fluences for each sample.

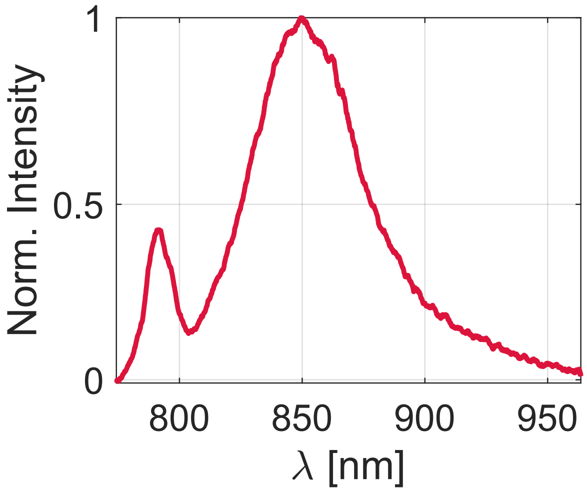

Figure S6: Spontaneous emission of the gain medium and the pump signal. The spectrum is averaged over several rows (k_y-axis) of the recorded spectrometer data and slightly smoothed for better visualization.

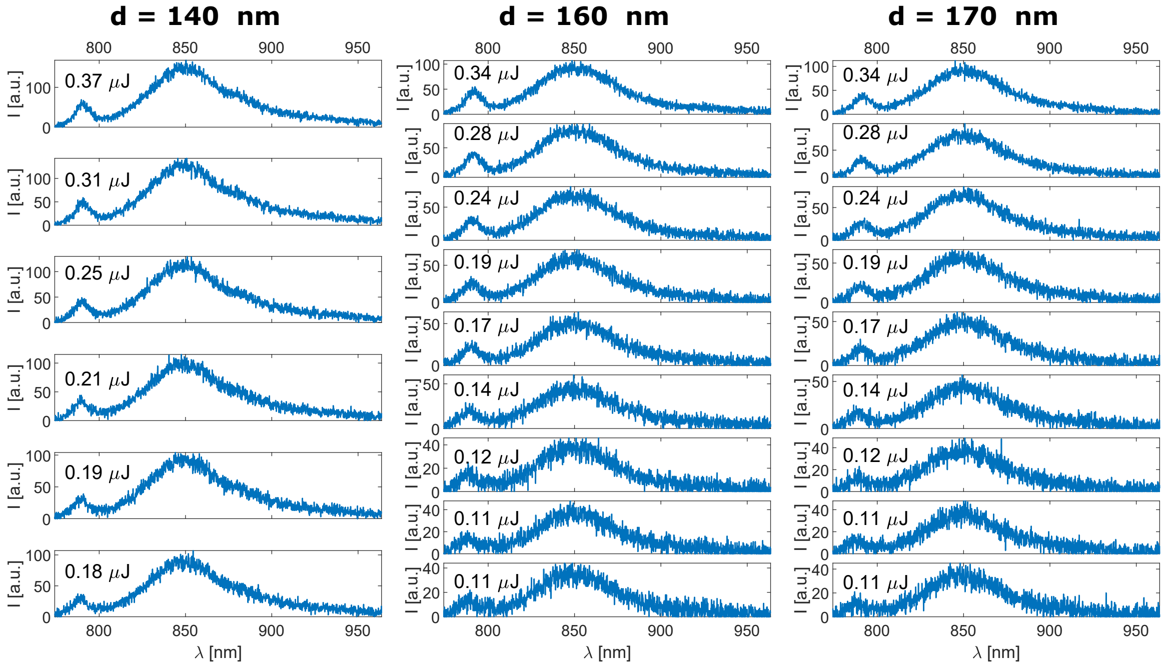

Figure S7: Recorded raw spectra below the lasing threshold of each sample. Corresponding pump fluence is marked next to each spectrum. Data shown here are plotted from a single row.

Section S6. Electric fields of identified lattice modes computed with T-matrix method

We used T-matrix simulations to identify the lasing modes in the system. [10, 11] Figures S8 and S9 show the computed fields in xy- and xz-planes for A_2'' and B_2' lattice modes.

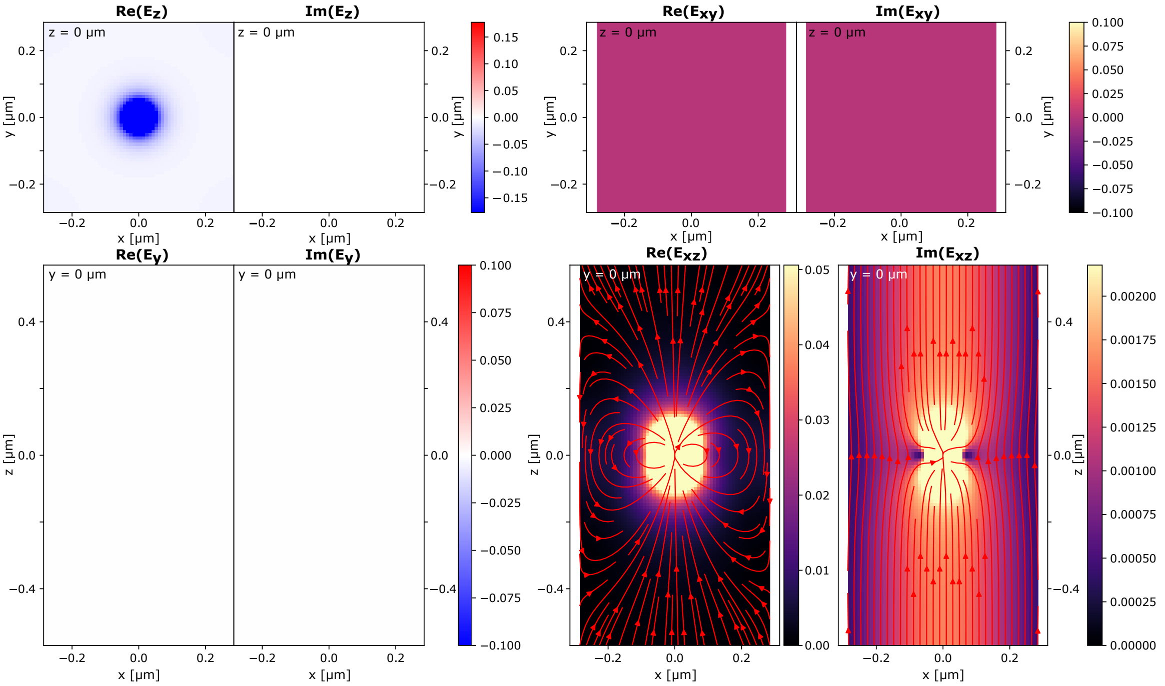

Figure S8: Electric field components of A_2'' lattice mode. Figures represent a single unit cell where nanoparticle is located in the center. Top row shows the xy-plane ($z = 0$) and bottom row shows the xz-plane ($y = 0$). Notably, in the xy-symmetry plane, the field contains only z-component.

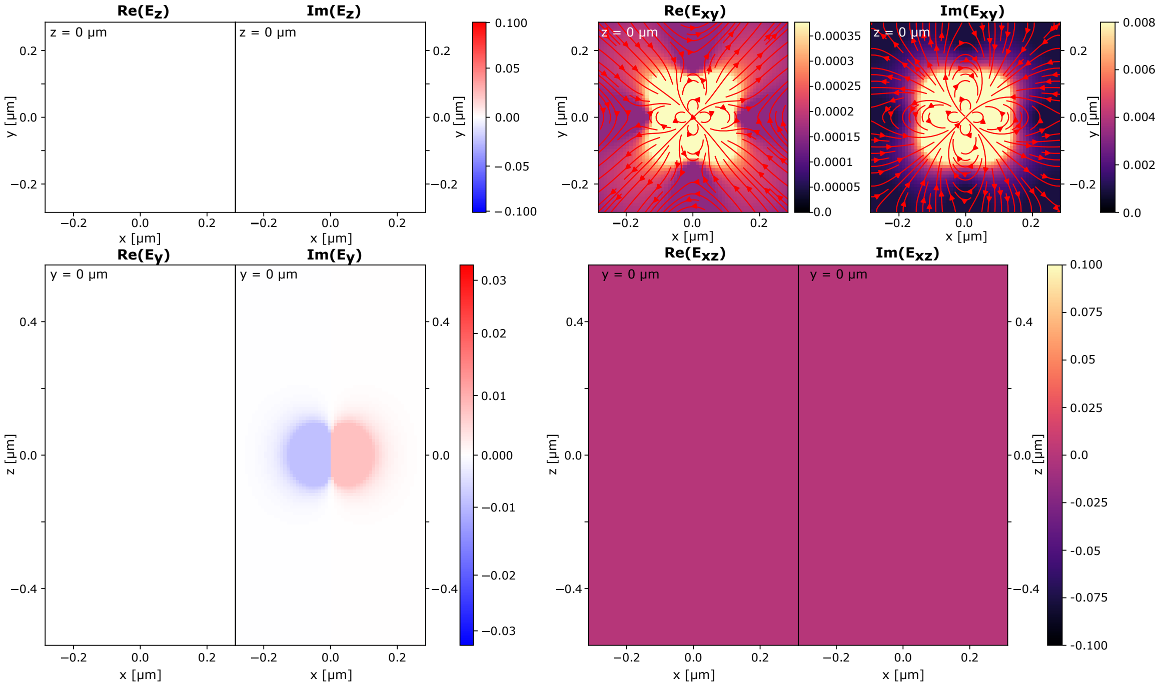

Figure S9: Electric field components of $B_2^{'}$ lattice mode. Top row shows the xy-plane ($z = 0$) and bottom row shows the xz-plane ($y = 0$). In contrast to the $A_2^{''}$ lattice mode, now the field contains x- and y-components in the xy-symmetry plane, while z-component remains zero.

Section S7. Processing of spatiotemporal coherence data

We use Fourier-transform method [12] in MATLAB to extract the degree of coherence (μ) variations from the measured fringe patterns. First, the fringe pattern is Fourier transformed by a two-dimensional discrete Fourier transform. The result is a two-dimensional matrix, which has two Fourier spectra, separated from the center by the fringe carrier frequency, and a background variation component at the center. We choose either of the Fourier spectra, and form a circular area around the peak value and set everything outside that area to zero. The chosen spectrum is translated to the center of the matrix, and we compute the inverse Fourier transform of it. The result is the μ, which is multiplied by two since half of the signal was cropped out in the Fourier space.

After this initial processing of the measured data, we could analyze the $|\mu|$ on different positions in the k-space and source plane. In both spaces, we analyzed a vertical slice at the center of the beam/lattice. Since in the far-field WFI the field was only flipped along horizontal axis, we investigate a vertical slice in both spaces. The slice was divided into smaller rectangles, and the values inside each rectangle were averaged. Each rectangle had a height of 12 pixels (0.028° and 1.2 μm)), and the width was 30 pixels (0.07°) in the k-space and 200 pixels (16 μm) on the source plane. The averaged rectangles are shown in Fig. S10 in both spaces. By taking the average on each time delay, we got the temporal evolution of the $|\mu|$ shown in the main manuscript.

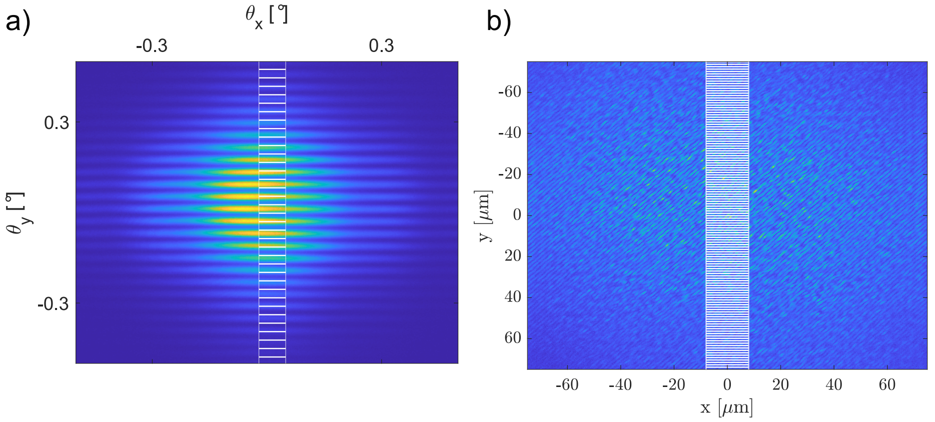

Figure S10: Area averaging for analysis of coherence properties in a) k-space and b) real-space. The rectangles have the same height in pixels (12) in both spaces, and the k-space image is zoomed in.

Fig. S11 demonstrates the resulting $|\mu|$ if the beam center is excluded from the area averaging. Notably, now the temporal beating is present everywhere along θ_y-axis. Thus, it is reasonable to include the beam center in the far-field analysis.

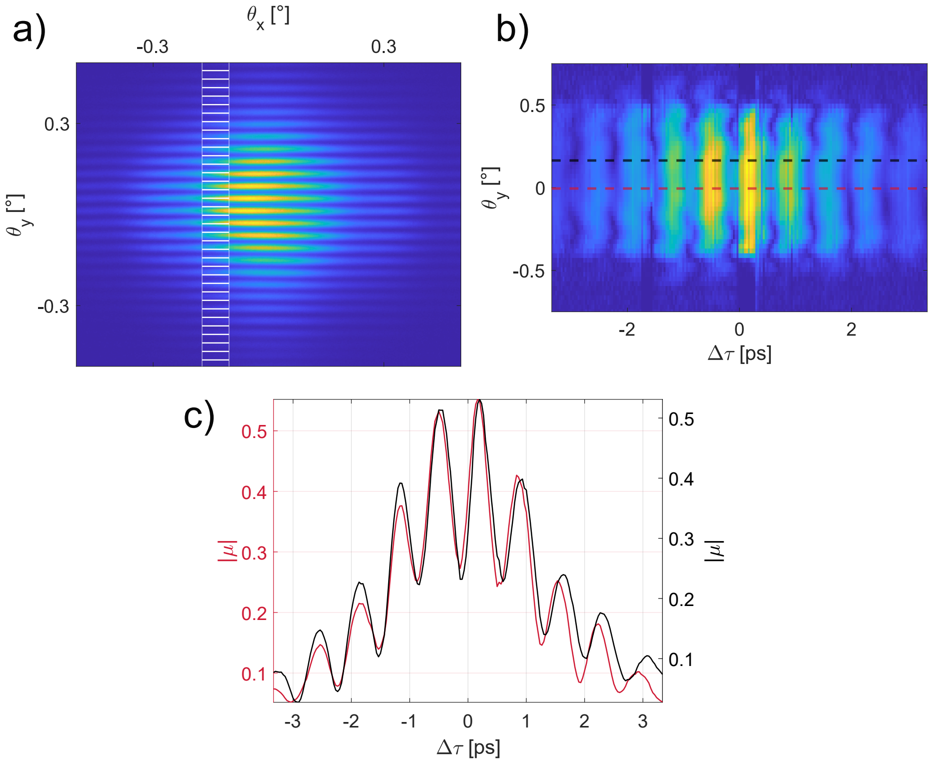

Figure S11: Demonstration of degree of coherence when the beam center is excluded from the calculation area. a) Chosen area for calculation. b) Resulting degree of coherence as a function of time. c) Line graphs of $|\mu|$ along black and red lines in b).

Fig. S12 demonstrates resulting $|\mu|$ if the averaging is done in the horizontal direction at the source plane. The results are similar compared to the vertical averaging, implying that the beating takes place on the edges of the lattice.

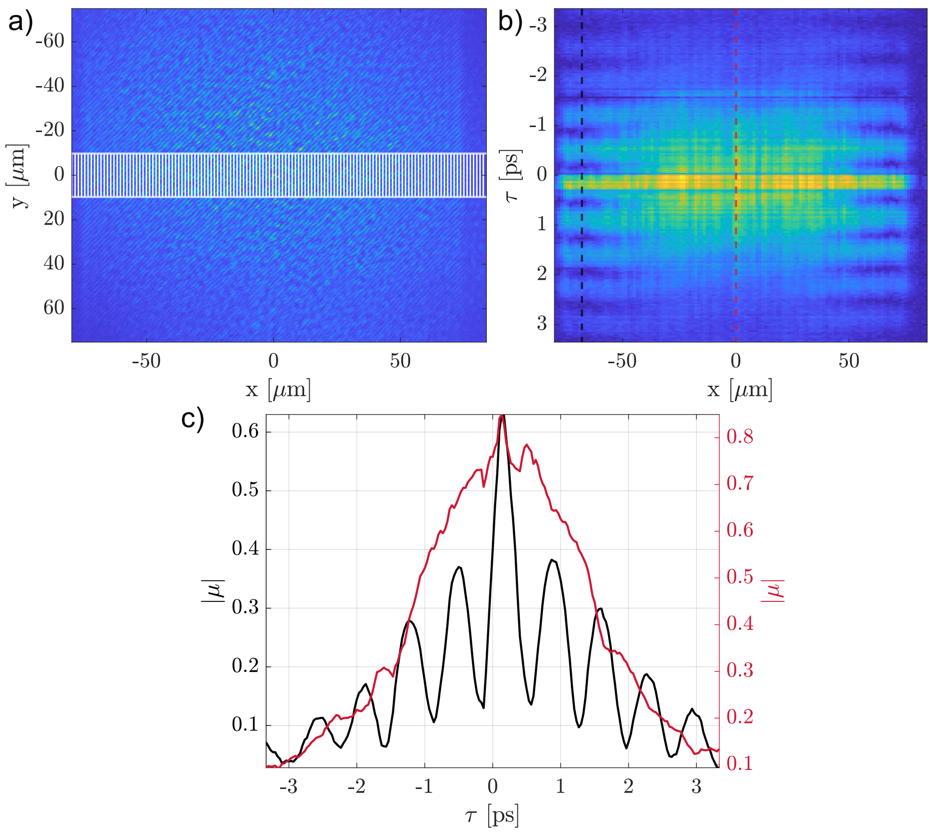

Figure S12: a) Horizontal area chosen for calculation. b) Resulting degree of coherence as a function of time. Note that now time axis is in $y-$direction, opposite to what is shown in the main manuscript. c) Line graphs of $|\mu|$ along black and red lines in b). The sample is the one with $d = 160$ nm.

Section S8. Additional measured FROG traces

Figure S13 shows measured FROG traces of d = 140 nm and d = 170 nm lattices. The traces contain summed SF intensity values over all relevant θ_y-angles, i.e., all angles that showed intensity above the noise level ($\sim$ 20 counts).

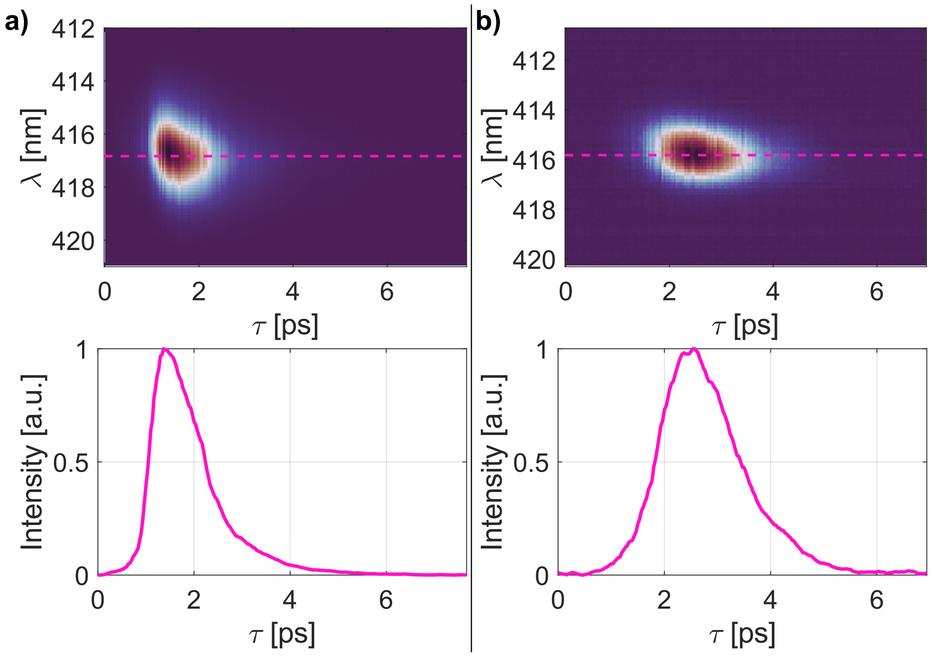

Figure S13: Measured FROG traces of a) $d = 140$ nm and b) $d = 170$ nm lattices.

The Gaussian fitting for the measured SF trace was done with the following function

$$f(x) = a \exp\left[-\left(\frac{x-b}{2c}\right)^2\right] + d, \tag{11}$$

where a is the height of the curve's peak, b is the position of the peak, c determines width of the curve, and d is an additional constant. x is the wavelength, which we obtain from the raw data. When we place the Gaussian function in the context of the measured data, a corresponds to the SF intensity of the mode, b is the central wavelength, c determines the spectral width, and d is for the background noise. We did two fits, one for both modes, for every measured time delay.

Section S9. FDTD simulations of lasing in an infinite plasmonic lattice

We performed FDTD simulations (Lumerical Inc.) with a built in four-level laser gain model to study the lasing in plasmonic lattices. The xz-view of the simulation geometry is depicted in Fig. S14(a). A single cylindrical Au NP was simulated with periodic boundary conditions in x- and y-directions and perfect matching layers in the z-direction. The NP had a diameter of 160 nm and thickness of 50 nm and the periodicity of the unit cell was $p_{x,y} = 570$ nm. The NP was overlaid with a 100 nm thick four-level gain material, where the level populations follow the rate equations given by [13]

$$\frac{dN_3}{dt} = -\frac{N_3(1-N_2)}{\tau_{32}} - \frac{N_3(1-N_0)}{\tau_{30}} + \frac{1}{\hbar\omega_{30}}\boldsymbol{E}\cdot\frac{d\boldsymbol{P}_{30}}{dt} \tag{12}$$

$$\frac{dN_2}{dt} = \frac{N_3(1-N_2)}{\tau_{32}} - \frac{N_2(1-N_1)}{\tau_{21}} + \frac{1}{\hbar\omega_{21}}\boldsymbol{E}\cdot\frac{d\boldsymbol{P}_{21}}{dt} \tag{13}$$

$$\frac{dN_1}{dt} = \frac{N_2(1-N_1)}{\tau_{21}} - \frac{N_1(1-N_0)}{\tau_{10}} - \frac{1}{\hbar\omega_{21}}\boldsymbol{E}\cdot\frac{d\boldsymbol{P}_{21}}{dt} \tag{14}$$

$$\frac{dN_0}{dt} = \frac{N_3(1-N_0)}{\tau_{30}} + \frac{N_1(1-N_0)}{\tau_{10}} - \frac{1}{\hbar\omega_{30}}\boldsymbol{E}\cdot\frac{d\boldsymbol{P}_{30}}{dt}, \tag{15}$$

where τ_{ij}, $i,j = 0,1,2,3$ is the time decay constant between levels i and j, N_i is the population density of level i, ω_{30} (ω_{21}) is the resonance frequency of absorption (emission) transition, $\boldsymbol{P}_{30,21}$ is the polarization density, and $\boldsymbol{E}$ is the electric field. The absorption and emission transitions are governed by the equations

$$\frac{d^2\boldsymbol{P}_{21}}{dt^2} + \gamma_{21}\frac{d\boldsymbol{P}_{21}}{dt} + \omega_{21}^2\boldsymbol{P}_{21} = \zeta_{21}(N_2 - N_1)\boldsymbol{E} \tag{16}$$

$$\frac{d^2\boldsymbol{P}_{30}}{dt^2} + \gamma_{30}\frac{d\boldsymbol{P}_{30}}{dt} + \omega_{30}^2\boldsymbol{P}_{30} = \zeta_{30}(N_3 - N_0)\boldsymbol{E}, \tag{17}$$

where $\gamma_{30,21}$ are the damping coefficients accounting for non-radiative losses and $\zeta_{30,21} = 6\pi\varepsilon_0 c^3/(\omega_{30,21}^2\tau_{30,21})$, where ε_0 is the vacuum permittivity and c is the speed of light. Equations (12)-(15) and (16)-(17) are coupled with the Maxwell-Ampere law solved by the FDTD algorithm.

The gain material parameters used in the simulation were $\omega_{21} = 2.1405 \cdot 10^{15}$ rad/s, $\omega_{30} = 2.3844 \cdot 10^{15}$ rad/s, $\gamma_{21} = 3.825 \cdot 10^{13}$ Hz, $\gamma_{30} = 3.26 \cdot 10^{14}$ Hz, $\tau_{30} = 1$ ns, $\tau_{32} = 10$ fs, $\tau_{21} = 1$ ns, $\tau_{10} = 10$ fs, and the electron population density $N = 1.5 \cdot 10^{25}$ m^{-3}. All electrons were assumed to be initially on the ground level N_0.

The structure was pumped with a normally incident circularly polarized plane wave pulse with temporal duration of 50 fs. The refractive index of the background was 1.52 + 0.01i and the refractive index of Au was taken from Ref. [14]. A symmetric, uniform mesh with maximum mesh step size of 5 nm x 5 nm x 2 nm in x-y-z directions was used around the nanoparticle in a region of 570 nm x 570 nm x 60 nm and a non-uniform mesh was used elsewhere.

Figure S14(b) shows the time- and wavelength resolved electric field combined from 40 different points located near the nanoparticle and inside the gain region. A narrow peak appears around 882 nm shortly after the pump pulse, indicating the onset of lasing. The data below the white dashed line is divided by a factor of 5 to better visualize the sample emission compared to the high intensity pump pulse at 790 nm.

Figure S14(c) left panel presents the time-integrated norm of the electric field in the xy-plane through the center of the NP at the lasing wavelength of 882 nm. The field exhibits a quadrupolar pattern with the plasmonic hot spots located along the diagonals of the NP. The field polarization, indicated by black arrows, matches the B_2' mode shown in Fig. 2d of the main manuscript. The corresponding charge density distribution in the right panel further confirms the quadrupolar nature of the lasing mode.

Figure S14(d) displays the spatial maps of the stimulated emission (left panel), the norm of the electric field (middle panel), and the population inversion (right panel) at two different time points: $t = 0.4$ ps (top row) and $t = 1$ ps (bottom row). The former corresponds to a time just after the pump pulse hits the structure, exciting electrons in the gain material to higher energy levels. The stimulated emission follows the pump field and the population inversion reaches high values outside the NP region. At $t = 1$ ps, after the pump has decayed, both the stimulated emission and electric field become concentrated at the plasmonic hot spot regions where lasing takes place. The population inversion deplates at the diagonals where lasing occurs and remains relatively high in regions where the electric field of the mode is weak.

We note that even though the lasing experiments with particle diameter $d = 160$ nm exhibit two lasing modes, the simulations show only a single dominant lasing mode. In Figure S14(b), the emission initially occurs over a wide bandwidth around 900 nm, but as the quadrupolar mode at 882 nm starts to lase, the emission at 900 nm rapidly diminishes. This behavior might arise due to gain competition and from the fact that the simulation models an infinite lattice, in which the quadrupolar mode experiences very low losses due to the absence of the sample edges. In addition, in the simulation the sample is pumped with normally incident light whereas in the experiments angled pump was used. The angled pump can favor the excitation of different modes compared to normally incident pump. Using an angled pump in the simulation would have increased the time- and memory consumption drastically.

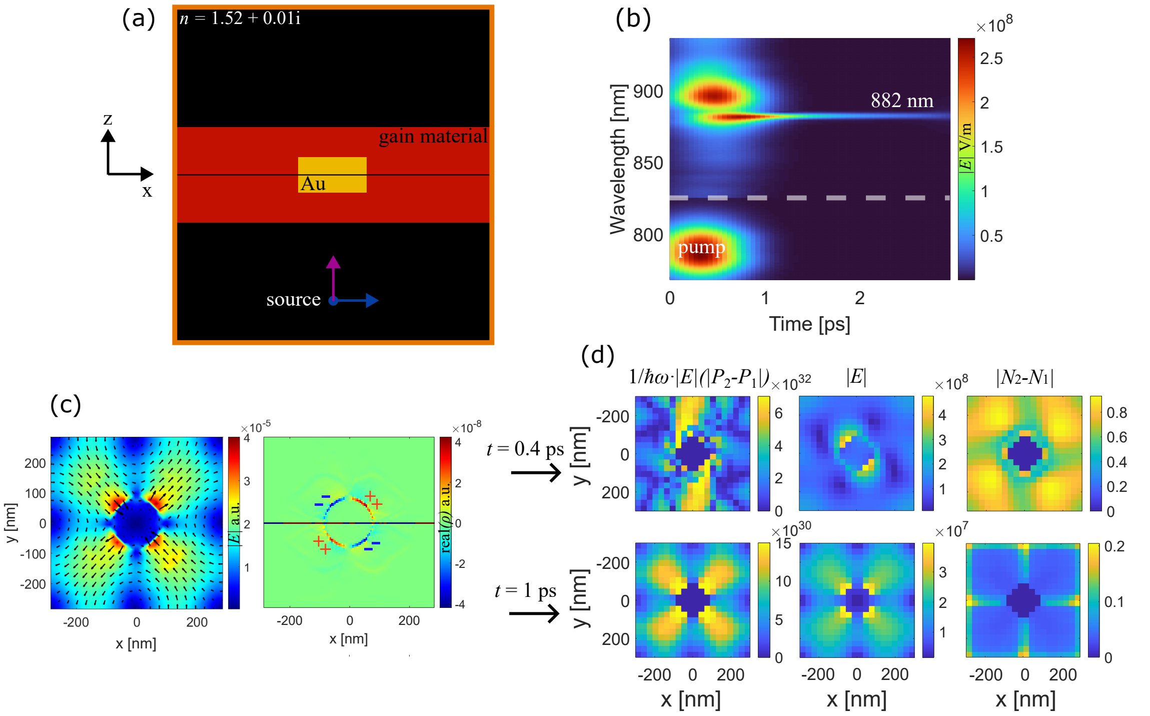

Figure S14: Lasing simulations with Lumerical FDTD. (a) xz-view of the simulation geometry. (b) Time- and wavelength-resolved electric field at the lattice plane. The data below the gray dashed line has been divided by a factor of 5 to better visualize the emission from the structure. (c) Time-integrated electric field (left) and the charge density distribution of the lasing qBIC-mode. (d) Spatial maps of stimulated emission (left), electric field (middle) and population inversion (right) at $t = 0.4$ ps (top row) and $t = 1$ ps (bottom row).

References

(1) Koivurova, M.; Partanen, H.; Lahyani, J.; Cariou, N.; Turunen, J. *Opt. Express* **2019**, *27*, 7738–7750.

(2) Halder, A.; Partanen, H.; Leinonen, A.; Koivurova, M.; Hakala, T. K.; Setälä, T.; Turunen, J.; Friberg, A. T. *Opt. Lett.* **2020**, *45*, 4260–4263.

(3) Turunen, J.; Halder, A.; Koivurova, M.; Setälä, T. *J. Opt. Soc. Am. A* **2022**, *39*, C214–C239.

(4) Koivurova, M.; Laatikainen, J.; Friberg, A. T. *Journal of the Optical Society of America A* **2024**, *41*, 615.

(5) Heikkinen, J. I.; Calpe, R.; Yrjänheikki, L.; Halder, A.; Moilanen, A. J.; Koivurova, M.; Hakala, T. K. *Laser Photonics Rev.* **2026**, *20*, e02215.

(6) Zhen, B.; Hsu, C. W.; Lu, L.; Stone, A. D.; Soljačić, M. *Phys. Rev. Lett.* **2014**, *113*, 257401.

(7) Asamoah, B. O.; Nečada, M.; Liu, W.; Heikkinen, J.; Mohamed, S.; Halder, A.; Rekola, H.; Koivurova, M.; Väkeväinen, A. I.; Törmä, P.; Turunen, J.; Setälä, T.; Friberg, A. T.; Shi, L.; Hakala, T. K. Finite Size Mediated Radiative Coupling of Lasing Plasmonic Bound State in Continuum, arXiv:2206.05011, 2022.

(8) Mohamed, S.; Wang, J.; Rekola, H.; Heikkinen, J.; Asamoah, B.; Shi, L.; Hakala, T. K. *Laser Photonics Rev.* **2022**, *16*, 2100574.

(9) Hakala, T. K.; Rekola, H. T.; Väkeväinen, A. I.; Martikainen, J.-P.; Nečada, M.; Moilanen, A. J.; Törmä, P. *Nat. Commun.* **2017**, *8*, 13687.

(10) Nečada, M.; Törmä, P. *Commun. Comput. Phys.* **2021**, *30*, 357–395.

(11) Nečada, M. QPMS Photonic Multiple Scattering Suite, https://qpms.necada.org, 2021.

(12) Takeda, M.; Ina, H.; Kobayashi, S. *Journal of the Optical Society of America* **1982**, *72*, 156.

(13) Chang, S.-H.; Taflove, A. *Opt. Express* **2004**, *12*, 3827–3833.

(14) *Handbook of Optical Constants of Solids*; Palik, E. D., Ed.; Academic Press: 1998; Vol. 1.
